# Soft, Flexible, and Stretchable Platforms for Tissue‐Interfaced Bioelectronics

**DOI:** 10.1002/advs.202521521

**Published:** 2026-02-03

**Authors:** Kento Yamagishi, Sunghoon Lee, Tomoyuki Yokota, Takao Someya

**Affiliations:** ^1^ Department of Electrical Electronic Engineering and Information Systems The University of Tokyo Bunkyo‐ku Tokyo Japan; ^2^ Thin‐Film Device Laboratory Wako Saitama Japan; ^3^ RIKEN Center for Emergent Matter Science (CEMS) Wako Saitama Japan; ^4^ Institute of Engineering Innovation Graduate School of Engineering The University of Tokyo Bunkyo‐ku Tokyo Japan

**Keywords:** bio‐integrated electronics, flexible electronics, stretchable electronics, tissue adhesives

## Abstract

Seamless integration of electronic systems with living tissues requires not only biocompatibility but also careful matching of mechanical properties across heterogeneous organs. This review clarifies the often‐conflated notions of “soft,” “flexible,” and “stretchable” electronics, and links these definitions to a tissue‐mechanics framework spanning brain, nerve, skin, myocardium, and visceral organs. Based on this framework, we outline general mechanical design principles—ultrathin structures, stretchable architectures, and bioadhesive interfaces—that enable deformable devices to conform to moving, curved surfaces. Recent advances are then organized into tissue‐targeted platforms, including imperceptible skin‐mounted nanosheet and nanomesh electrodes, haptic and neural interfaces for bidirectional communication, and wet‐organ adhesive systems for cardiac and gastrointestinal applications. We further highlight emerging material systems such as liquid metal–based conductors and biodegradable transient electronics, which respectively extend mechanical adaptability and introduce time‐programmed disappearance to reduce surgical burden. Across these topics, the review distills unifying design rules for matching modulus, adhesion, and strain tolerance to specific biological environments, positioning soft, tissue‐interfaced bioelectronics as a coherent toolbox that bridges wearable, implantable, and transient formats for future healthcare technologies.

## Graphical abstract

Bio‐integrated electronics provide mechanically compliant and stable interfaces with soft biological tissues. Representative applications include neural interfaces, wet‐organadhesive electronics, and skin‐interfaced devices. E represents Young´s modulus and ε represents strain.

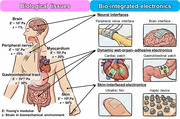

## 1 Introduction

Advances in microfabrication, materials science, and device engineering have rapidly transformed electronic systems into compact, lightweight, and high‐performance technologies, driving their widespread adoption as portable, wearable, and implantable devices. Building on this trend, a new paradigm of electronics has emerged, emphasizing softness, flexibility, and stretchability to achieve seamless integration with biological tissues [1, 2, 3]. Although these mechanical properties are often used interchangeably, they represent distinct physical concepts that must be clearly defined to minimize mismatch with living systems. Soft materials are characterized by a low elastic modulus and tissue‐like conformity, which enable intimate contact with biological surfaces. Flexible materials are defined by their ability to bend without fracture, a property governed by low flexural rigidity, as observed in thin metal foils and polymer films. Stretchable materials, in contrast, can withstand large tensile strains and recover their original shape, exemplified by elastomers and hydrogels. Distinguishing among these categories is essential for guiding material choices and integration strategies tailored to specific biomedical applications.

**FIGURE 1:**
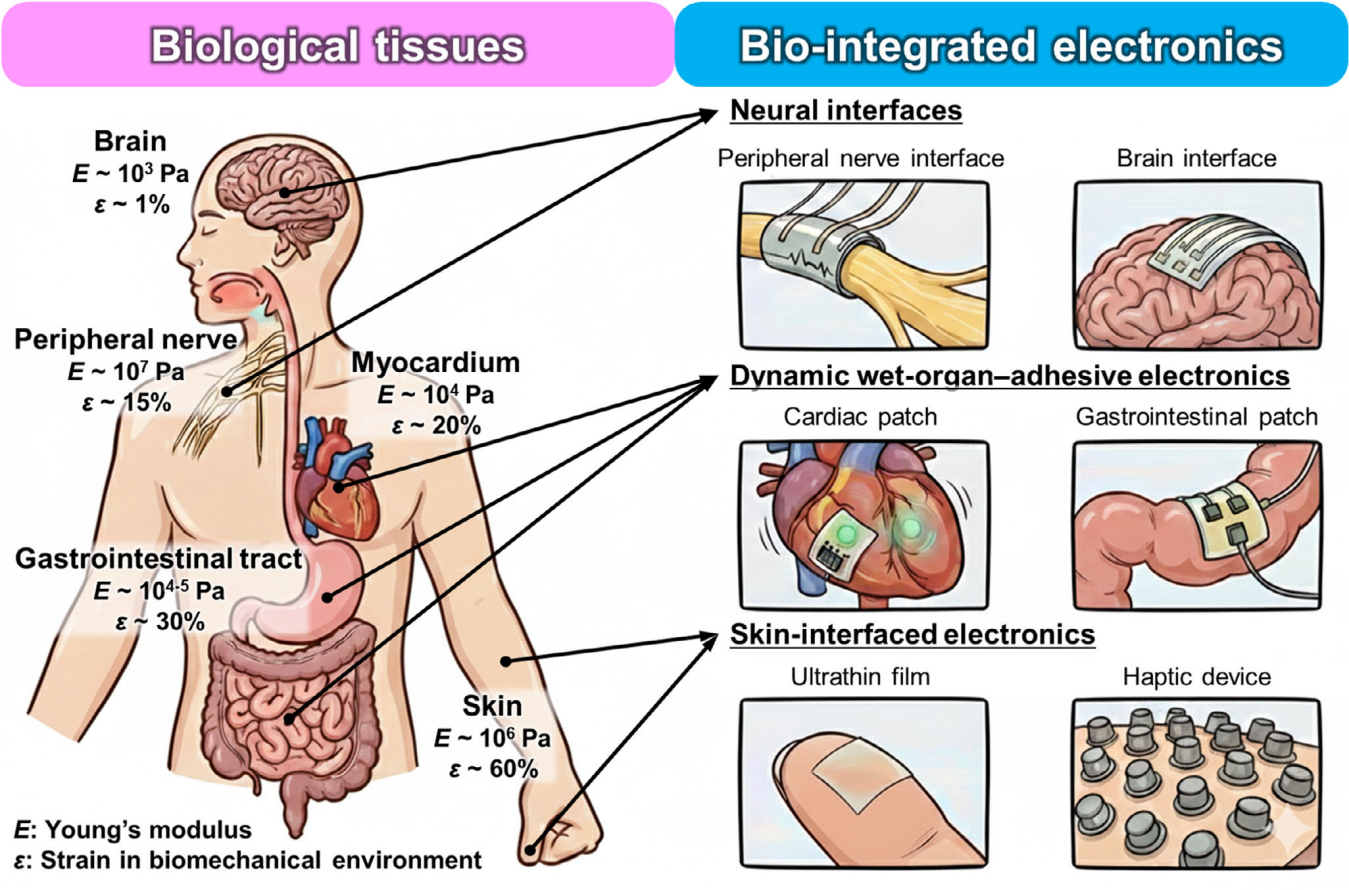
Tissue‐interfaced bioelectronics achieve seamless integration with living systems by matching their mechanical properties to those of targeted biological tissues. The left panel schematically summarizes representative tissues—including brain, peripheral nerve, myocardium, gastrointestinal tract, and skin—together with typical ranges of Young's modulus *E* and physiological strain ε, highlighting the wide span from ultra‐soft neural tissue to highly stretchable skin. The right panel illustrates three major classes of bio‐integrated electronics organized by anatomical target: neural interfaces (peripheral nerve [156] and brain [232] interfaces), dynamic wet‐organ–adhesive electronics (cardiac [189] and gastrointestinal [118] patches), and skin‐interfaced electronics (ultrathin epidermal films [134] and haptic devices [138]). Arrows indicate how each device class is mechanically and anatomically mapped to its corresponding tissue environment, emphasizing that appropriate combinations of softness, flexibility, and stretchability are essential to maintain stable coupling, minimize mechanical mismatch, and preserve normal biomechanical function.

Within the same organism, biological tissues differ markedly in their mechanical properties, surface energies, and strain limits, reflecting their distinct anatomical locations and physiological roles [4]. For instance, the brain is extremely soft and fragile, while the skin exhibits exceptionally high stretchability and conformability. In contrast, tendons and cartilage are stiffer and less extensible, designed to sustain mechanical loads, whereas muscles and internal organs remain soft and compliant. Because of these differences, no single material or structural design can universally interface with all tissues across the body. Instead, successful bioelectronic systems must be customized for each organ, carefully matching the modulus, adhesion characteristics, and deformation behavior to the surrounding biological environment. The tissue‐specific mechanical landscape of the body—and the corresponding families of bio‐integrated electronic platforms tailored to the brain, peripheral nerves, myocardium, gastrointestinal tract, and skin—is illustrated schematically in Figure 1. This organ‐aware design approach marks a shift away from generic flexible electronics toward function‐driven, organ‐adaptive systems that can maintain long‐term stability and minimize physiological disturbance across the body's diverse mechanical environments.

Beyond medical and healthcare applications, bio‐integrated electronic systems hold great potential in diverse fields such as sports science [5, 6, 7], virtual and augmented reality [8], and human motion augmentation [9]. Although the required operating time and mechanical durability vary depending on the intended use, a shared design philosophy prevails across these domains: devices should adhere to the body with minimal discomfort while maintaining long‐term stability and safety. To realize this vision, researchers have developed complementary strategies focusing on both the substrate and the conductive elements. Substrate‐oriented approaches aim to achieve mechanical matching and conformal adhesion to tissues by employing soft elastomeric materials or hydrogels whose Young's moduli are comparable to those of biological organs [10]. An alternative strategy is to reduce the substrate thickness to the micrometer scale, which markedly lowers flexural rigidity, enhances conformal contact with tissue microtopography, and thereby promotes effective adhesion through van der Waals interactions without the need for additional adhesives [11, 12]. Conductor‐oriented approaches ensure electrical functionality under large deformations through either structural engineering—such as serpentine interconnects [13, 14, 15], mesh [16, 17, 18] or kirigami [7, 19, 20, 21, 22] architectures, and controlled microcracks [23, 24]—or the use of intrinsically deformable conductive materials, including nanocomposites [25, 26], conjugated polymers [27, 28, 29, 30], hydrogels [31, 32], and liquid metals [33, 34]. The integration of these design philosophies enables the creation of electronic systems that maintain mechanical integrity, electrical performance, and biocompatibility during continuous operation within dynamic biological environments.

This review focuses on recent advances in soft, tissue‐interfaced bioelectronics, spanning fundamental mechanics to clinical implementation. To provide a coherent framework, the article is organized into four complementary layers: (i) general mechanical design principles and strategies for achieving device‐level stretchability, (ii) tissue‐targeted platforms for skin, neural, and internal‐organ interfaces, (iii) next‐generation material systems, including liquid metal–based conductors and transient, bio‐degradable electronics, and (iv) representative applications in which these technologies are already being used in clinical settings. Within this structure, we distill unifying design concepts, compare representative implementations, and propose guidelines for realizing truly tissue‐integrated electronic systems that operate in long‐term harmony with biological environments.

## 2 Mechanical Design Principles for Bioelectronic Integration

**FIGURE 2:**
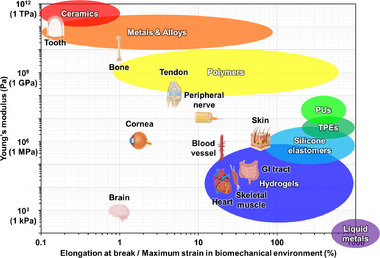
Mechanical properties of biological tissues and representative electronic materials. Young's modulus and maximum strain of various biological tissues compared with commonly used materials in electronic device fabrication, including ceramics, metals, alloys, polymers, elastomers, hydrogels, and liquid metals. PU: polyurethane; TPE: thermoplastic elastomer; GI tract: gastrointestinal tract. Reproduced with permission [36]. Copyright 2024, Springer Nature.

Biological tissues exhibit an extraordinary diversity of mechanical properties, spanning over ten orders of magnitude in Young's modulus depending on their composition and physiological role. Figure 2 summarizes typical orders of magnitude for the Young's modulus and extensibility of various tissues and materials, emphasizing illustrative ranges rather than precise measurements, since the apparent modulus of a given tissue can vary substantially with measurement technique, loading conditions, and anatomical location [35, 36]. Tooth enamel and bone constitute the stiffest biological tissues: enamel exhibits elastic moduli reaching several tens of GPa and approaching 100 GPa (10^1^ ^1^ Pa) [37], while bone is comparatively softer, with elastic moduli of approximately 10–20 GPa (10^1^ ^0^ Pa) [38]. Next, tendons and cartilage remain mechanically robust but are less stiff than mineralized tissues, displaying elastic moduli spanning from approximately tens of MPa to the lower GPa range (10^7^–10^9^ Pa) [39, 40]. Soft connective and surface tissues—including skin, cornea, and peripheral nerves—exhibit substantially lower stiffness, typically in the range of hundreds of kPa to tens of MPa (10^5^–10^7^ Pa) [41, 42, 43]. In hollow organs, smooth muscle layers embedded in a compliant collagen–elastin matrix constitute the primary load‑bearing component in both blood vessels and the gastrointestinal (GI) tract. In blood vessels, vascular smooth muscle within the arterial wall behaves as a nonlinearly elastic material whose passive elastic modulus is typically on the order of 10^5^ Pa, and active contraction can transiently increase the apparent wall stiffness by several fold, thereby broadening the mechanical operating window of the vessel wall [44]. In the GI tract, circumferential and longitudinal smooth muscle layers organized in a collagen–elastin‑rich wall generally exhibit lower passive elastic moduli, often in the range of 10^3^–10^4^ Pa, and active smooth muscle contraction similarly elevates the effective wall stiffness by several fold over this softer baseline, expanding the usable mechanical range for luminal transport and motility [45, 46]. Skeletal muscle exhibits passive elastic moduli on the order of 10–50 kPa at the single‑fiber or cellular scale, with apparent stiffness increasing markedly upon stretching or during active force generation, consistent with its role in producing large joint excursions and rapid shortening–lengthening cycles [47, 48]. In contrast, ventricular myocardium is of a comparable but often higher stiffness, with passive elastic moduli frequently reported around 5–20 kPa at low strain and increasing to several tens of kPa or more under physiological loading, reflecting the need to withstand repetitive pressure–volume changes during the cardiac cycle while still permitting efficient pumping [49]. The brain represents the softest tissue, with elastic moduli of only a few kPa (10^3^ Pa), underscoring the extreme mechanical vulnerability and specialized protection required for neural structures [50].

In addition to stiffness, tissues differ markedly in their extensibility. Skin and muscle can accommodate large tensile strains, often on the order of several tens of percent; in vivo measurements around joints indicate that skin experiences reversible strains of up to 40%–45% during physiological motion [51], while in vitro tensile tests show that excised human skin fails at average strains of 50%–60%, with localized strain concentrations approaching ∼70% immediately before rupture [52]. In the GI tract, the wall also undergoes large deformations, but in a strongly organ‐, layer‐, and direction‐dependent manner that reflects its specialized roles in transport, mixing, and storage. Systematic studies of GI biomechanics show that the esophagus, stomach, small intestine, and large intestine can sustain passive circumferential strains of several tens of percent under physiological pressures, with even larger distension in reservoir segments such as the stomach and rectum during maximal filling [45, 46]. Superimposed on this compliant background, the muscularis propria generates active, anisotropic contractions that add cyclic circumferential and longitudinal strains of roughly a few to ∼10% during peristalsis, enabling coordinated bolus transport and controlled emptying while preserving the mechanical integrity of the layered wall. Similarly, skeletal muscle can undergo substantial but functionally constrained tensile strains during physiological movement, with in vivo measurements around major joints typically reporting passive muscle‑tendon or fascicle length increases of roughly 10%–30% within the normal range of joint motion, whereas experimental studies of actively lengthening muscle indicate that larger active strains of about 20%–30% (relative to resting or optimal length) are sufficient to induce marked structural disruption and persistent reductions in maximal isometric force, consistent with strain‑induced injury mechanisms [47, 48, 53]. The myocardium exhibits more moderate but tightly regulated deformation; in vivo speckle‐tracking and other imaging studies indicate that cardiac muscle undergoes physiological strains of approximately 15%–20% in the longitudinal direction and 20%–30% circumferentially during the cardiac cycle, reflecting large yet reversible cyclic deformation associated with contraction and relaxation [54]. Blood vessels also exhibit considerable extensibility. Large elastic arteries such as the aorta commonly undergo cyclic circumferential strains on the order of about 10%–20% over a cardiac cycle under normotensive conditions, whereas smaller muscular arteries and arterioles, or arteries exposed to hypertensive pressures, can experience larger cyclic or mean circumferential strains, often approaching or exceeding 20%–30% depending on vessel size, location, and loading history [44, 55, 56, 57]. Peripheral nerves also show appreciable but limited extensibility compared to other soft tissues. In vivo measurements indicate that nerves around joints can undergo longitudinal strains on the order of 10%–20% during physiological movements, while ex vivo tensile tests report failure strains typically around 20%–30%, at which point epineurial and perineurial collagen networks are fully recruited, and rupture occurs [43, 58]. Tendons are much less extensible than most soft tissues, typically operating within a narrow physiological tensile strain range of only 3%–6% and failing at strains of roughly 8%–15%, which underscores their limited safety margin between normal loading and rupture [59]. The cornea undergoes only modest physiological deformation, with normal loading typically producing local strains of just a few percent (1%–10%) [60, 61]. The brain parenchyma experiences even smaller cardiac‐induced deformations, with regional principal strains in healthy subjects generally on the order of ∼1% or less [62]. Bone deforms minimally under everyday loads, with typical functional cortical bone strains around 0.1%–0.3% and yielding or failure occurring near 0.7%–1.5% strain [63]. Taken together, these results highlight the substantial diversity in physiological strain tolerance among different tissues within the human body, emphasizing the importance of tailoring material properties and device mechanics to the specific target tissue.

In contrast, most conventional conductive materials, such as metals and alloys, have elastic moduli of 10^9^–10^1^ ^2^ Pa—many orders of magnitude greater than that of biological tissues—resulting in pronounced mechanical mismatch. While polymeric and elastomeric materials can cover a wide portion of the biological modulus spectrum (10^4^–10^8^ Pa) and thus serve as suitable structural substrates, they are intrinsically insulating. Hydrogels, despite offering excellent softness and water content comparable to tissues, also exhibit poor electrical conductivity. Consequently, a fundamental mechanical–electrical trade‐off arises: materials that are mechanically compatible with living tissues often lack sufficient conductivity, whereas conductive materials tend to be mechanically rigid. Liquid metals uniquely overcome this dilemma by combining metallic‐level conductivity (∼10^6^ S m^−^^1^) with a modulus in the tens to hundreds of pascals range, close to that of soft matter, making them promising candidates to bridge the mechanical gap between rigid electronics and soft biological systems [34].

**FIGURE 3:**
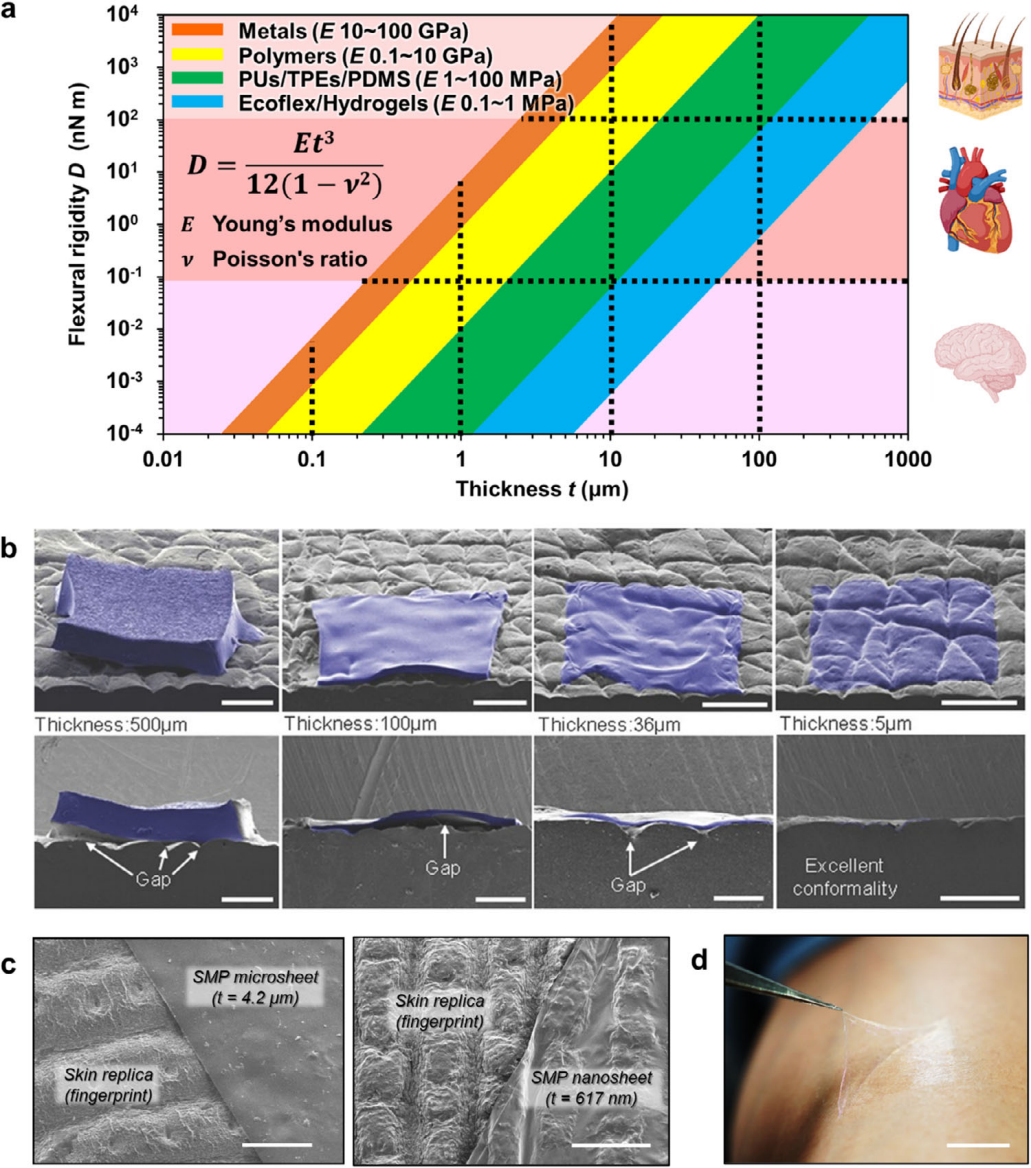
Flexural rigidity and conformability of thin films for bio‐integrated electronics. (a) Relationship between film thickness and flexural rigidity for materials with different Young's moduli. Reproduced with permission [288]. Copyright 2025, Wiley‐VCH. (b) Ecoflex (E = 69 kPa) thin films laminated on a silicone replica of the skin surface. Reproduced with permission [69]. Copyright 2013, Wiley‐VCH. (c) Left: Scanning electron microscope (SEM) image of a 4.2 µm‐thick shape‐memory polymer (SMP) microsheet (*T*_g_ = 25°C) attached to a silicone‐based skin replica. Right: SEM image of a 617 nm‐thick SMP nanosheet (*T*_g_ = 25°C) conforming to the same replica. The scale bars are 5 µm. Reproduced with permission [70]. Copyright 2019, American Chemical Society (ACS). (d) Photograph of a ∼200 nm‐thick poly(D,L‐lactic acid) (PDLLA) nanosheet attached to human skin. The scale bar is 1 cm. Reproduced with permission [12]. Copyright 2019, Royal Society of Chemistry (RSC).

When bioelectronic devices are laminated onto tissues, intimate adhesion is critical for both signal fidelity and mechanical stability. A key design parameter governing conformability is the flexural rigidity (*D*), which depends not only on the elastic modulus (*E*) but also strongly on the film thickness (*t*), following the cubic scaling relationship (Equation 1): [12, 64]

(1) $$D=\frac{Et^{3}}{12\left(1-\nu^{2}\right)}\;$$

where ν is Poisson's ratio, typically ranging from 0.3 to 0.5 for most metals and polymers. This strong thickness dependence means that even relatively stiff materials can behave as soft, tissue‐compatible films when made sufficiently thin. As shown in Figure 3a, typical metals (*E* ≈ 10–100 GPa) and polymers (*E* ≈ 0.1–10 GPa) are intrinsically stiff, yet their flexural rigidity can be reduced by several orders of magnitude simply by decreasing the film thickness from the micrometer to the nanometer range. When the thickness reaches a few hundred nanometers, their flexural rigidity becomes comparable to that of soft tissues such as the brain (E ≈ 0.1–16 kPa), enabling conformal contact even with highly compliant surfaces [16, 65]. In general, a 1–10 µm‐thick polymer film exhibits a flexural rigidity similar to that of internal organs such as the heart or liver, while tens of micrometers correspond to epidermal tissue. Conversely, submicrometer films (< 1 µm) can match or even fall below the stiffness of brain slices or mucosal tissue, entering the mechanical regime of ultra‐soft matter. These relationships demonstrate that, by tuning thickness alone, it is possible to align the mechanical behavior of conventional electronic materials with that of a wide range of biological tissues, ensuring intimate adhesion and minimizing interfacial stress.

Reducing film thickness not only lowers flexural rigidity but also enhances effective adhesion by promoting intimate conformal contact with the complex microtopography of biological tissues [1, 3, 66]. When the flexural rigidity becomes sufficiently low to accommodate surface roughness and curvature, the interface transitions from partial to full conformal contact. Conformal contact is governed by a balance between the bending (and stretching) energy required for the thin film to follow the substrate topography and the gain in interfacial adhesion energy, modulated by the amplitude and wavelength of tissue roughness. In the context of epidermal electronics, scaling analyses and finite‐element studies have shown that decreasing the film thickness (thereby reducing bending energy) and matching the roughness length scales of skin enables a transition from partial to fully conformal contact under a given adhesion energy [66, 67, 68]. Once equilibrium is achieved, the device forms a continuous and energetically favorable interface that adheres spontaneously through van der Waals interactions, eliminating the need for adhesives. This principle—often summarized as “thinner is more adhesive”—serves as a fundamental mechanical design guideline for ultrathin electronic systems that achieve mechanical comfort, interfacial stability, and seamless integration with the body.

As shown in Figure 3b, elastomeric materials such as Ecoflex exhibit excellent adhesion and follow skin wrinkles when thinned to only a few micrometers [69]. Likewise, polymer films with moduli in the tens of megapascals to gigapascal range, which are otherwise mechanically stiff, can achieve remarkable conformability and adhesion when fabricated with submicrometer thicknesses (Figure 3c) [70]. In addition, we have shown that biodegradable polylactic acid (PLA) nanosheets with a thickness of ∼200 nm adhere tightly to skin without any adhesives (Figure 3d), demonstrating that extreme thinning provides a universal route to achieving conformability and stable tissue integration [12].

In summary, the mechanical integration of bioelectronic systems relies on a delicate interplay between material properties, structural geometry, and interfacial mechanics. Through systematic modulation of elastic modulus, film thickness, and flexural rigidity, even intrinsically rigid materials can achieve tissue‐like conformity and stable adhesion across a wide range of biological environments. This foundation has established the essential mechanical framework for biointegration—where flexibility and softness are not merely aesthetic features but functional necessities that ensure long‐term stability, biocompatibility, and signal fidelity. Recent progress in the field has highlighted that true mechanical harmony with living tissues requires not only thin and compliant substrates but also adaptive interconnects and conductive components capable of enduring large and repetitive deformations without failure [71, 72]. Such insights define the transition from purely flexible to truly stretchable electronics, in which mechanical robustness and electrical reliability must coexist under continuous strain. The following section therefore, explores strategies to achieve this goal—focusing on material innovations, structural engineering approaches, and hybrid system architectures that enable electronic devices to stretch, twist, and deform in concert with the human body while maintaining functional integrity.

## 3 Strategies for Stretchable Electronics

**FIGURE 4:**
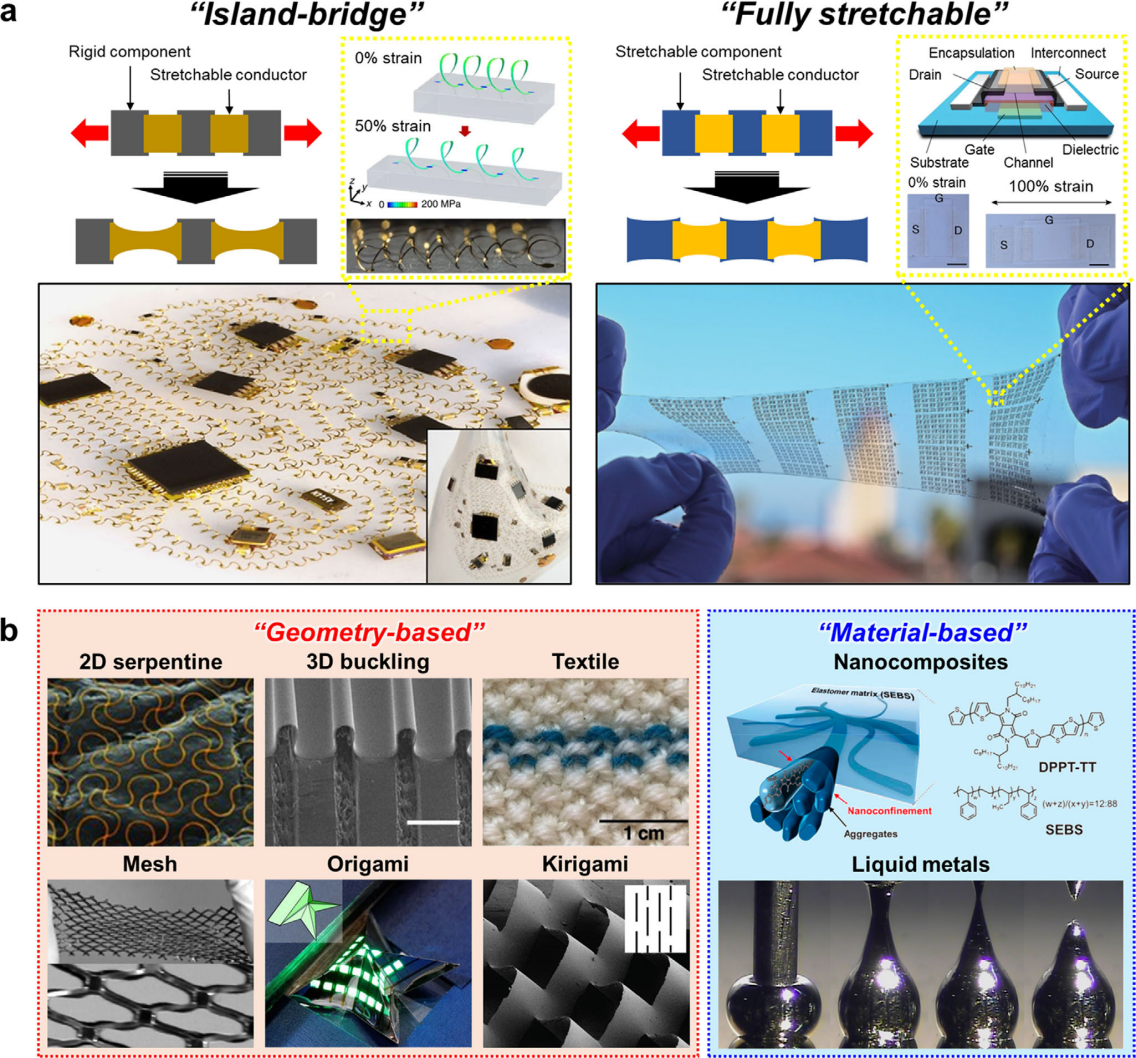
Strategies for stretchable electronics. (a) Two principal strategies: left, the island–bridge configuration where rigid components are connected by soft interconnects; right, the fully stretchable approach where all components, including conductors and active devices, are deformable. Left: Reproduced with permission [77]. Copyright 2017, Springer Nature. Right: Reproduced with permission [79]. Copyright 2024, Springer Nature. (b) Two complementary approaches for stretchable conductors: left, geometry‐based structural designs including 2D serpentine [13] (Reproduced with permission. Copyright 2013, Wiley‐VCH), 3D buckling [83] (Reproduced with permission. Copyright 2016, Springer Nature), textile [102] (Reproduced with permission. Copyright 2024, Springer Nature), mesh [17] (Reproduced with permission. Copyright 2005, National Academy of Sciences (NAS)), origami [105](Reproduced with permission. Copyright 2021, Springer Nature), and kirigami [22] (Reproduced with permission. Copyright 2015, Springer Nature); right, material‐based innovations including nanocomposites [30] (Reproduced with permission. Copyright 2017, American Association for the Advancement of Science (AAAS)) and liquid metals [33] (Reproduced with permission. Copyright 2008, Wiley‐VCH).

To realize electronic systems that can accommodate large deformations while maintaining stable performance, two principal strategies have been established (Figure 4a). Both approaches typically employ highly stretchable elastomeric substrates as the mechanical support. The first is the island–bridge strategy, in which rigid functional components such as transistors, diodes, or light‐emitting diodes (LEDs) are miniaturized into discrete “islands” and interconnected by serpentine or wavy stretchable conductors that act as “bridges.” [15, 73, 74, 75, 76, 77] This design localizes strain within the compliant interconnects, thereby protecting the fragile active components while allowing the overall system to stretch. The second approach is the fully stretchable strategy, where not only the substrate and wiring but also the active devices themselves are fabricated from intrinsically stretchable materials, such as organic semiconductors, conductive polymers, or liquid metals [78, 79, 80]. While the island–bridge architecture has demonstrated high‐performance integrated circuits and displays with reliable stretchability, the fully stretchable approach offers the potential for seamless mechanical compliance across the entire system. Together, these complementary strategies define the foundation for designing next‐generation stretchable bioelectronics.

Stretchable interconnects and devices can be realized through two complementary approaches: geometry‐based structural designs and material‐based innovations (Figure 4b). In the geometry‐based approach, mechanical compliance is imparted by engineering structural layouts that redistribute strain. Two‐dimensional serpentine patterns, fabricated by conventional photolithography or laser cutting, can be transferred onto elastomer substrates to create stretchable wiring [2, 13, 14, 81]. Three‐dimensional buckling structures arise when thin films are laminated onto pre‐stretched elastomers and subsequently released, and introducing predefined surface patterns enables periodic buckling that greatly improves durability under repeated stretching [82, 83, 84, 85, 86, 87, 88, 89, 90, 91, 92]. Fiber‐ and textile‐based geometries—such as woven, knitted, and braided fabrics—provide macroscopic stretchability and conformability through loop rotation and yarn reconfiguration rather than intrinsic fiber elongation, offering an alternative route to stretchable and breathable electronic fabrics suitable for large‐area, body‐mounted applications [93, 94, 95, 96, 97, 98, 99, 100, 101, 102]. Mesh, origami, and kirigami architectures further expand the design space for stretchable electronics, allowing 2D films to undergo large reversible deformations, especially when integrated with elastomers that provide restoring forces [7, 17, 19, 20, 21, 22, 103, 104, 105].

In parallel to structural design approaches, material‐based strategies focus on developing intrinsically deformable conductors and semiconductors. Intrinsically stretchable systems based on conductive polymers [27, 29, 30], ionic conductors [106, 107, 108], and hydrogels [31, 32] have been extensively reported, offering soft mechanical properties and good biocompatibility; however, these materials typically exhibit limited electrical conductivity, and their transport characteristics often change markedly when stretched, which remains a key bottleneck for reliable operation under large deformation. Nanocomposites consisting of elastomer matrices filled with conductive fillers—such as carbon nanotubes, graphene, or metallic nanoparticles—enable tunable conductivity and compliance, although their performance is often limited by incomplete percolation and loss of conductive pathways under strain [27, 109, 110, 111]. Recent advances include hybrid composites that combine multiple filler types to enhance carrier transport, as well as post‐processing treatments to improve dispersion and interfacial coupling; nevertheless, these approaches still face trade‐offs between stretchability, conductivity, and stability, motivating the search for alternative material platforms such as liquid metals that can complement, rather than replace, these earlier classes of intrinsically deformable conductors [112, 113].

Another promising route involves incorporating liquid metals such as eutectic gallium–indium (EGaIn) and Galinstan into soft matrices [33]. These materials provide metallic‐level conductivity (∼10^6^ S/m) while behaving as ultra‐soft fluids, uniquely combining high electrical performance with extreme mechanical deformability. Liquid metals not only provide high conductivity but also uniquely enable reconfigurable, self‐healing, and dynamically adaptive electronic systems because their metallic phase remains fluid at operating temperatures, allowing conductive pathways to flow, reshape, and reconnect under modest stimuli [114, 115, 116, 117, 118, 119]. Their fluidity makes it possible to reconfigure interconnects and circuit layouts through simple mechanical deformations (such as stretching, folding, and channel deformation) or through external fields and pressure. When liquid‐metal traces are fractured or segmented, the droplets can autonomously merge under capillary and confinement forces to restore electrical continuity—behavior that is extremely difficult to realize with conventional solid metals or static conductive composites. Wearable and implantable implementations of such liquid metal–based soft devices are discussed in detail in Section 7.

Building on these strategies, the following sections introduce representative device demonstrations that apply these design principles, categorized according to their target applications. All of these examples share the common goal of minimizing the mechanical mismatch between biological tissues and electronic systems, thereby enabling truly bio‐integrated platforms. As a starting point, we first focus on ultrathin skin electrodes, an area to which our group has devoted significant efforts, and which exemplifies how extreme mechanical compliance can enable stable, long‐term epidermal sensing.

## 4 Skin‐Interfaced Electronics

Building on the general mechanical design principles and stretchability strategies outlined in Sections 2 and 3, this section focuses on electronic systems that interface directly with skin. For skin‐mounted platforms, key device requirements include ultralow flexural rigidity, modulus matching to the epidermis, breathable and irritation‐free contact, and stable electrical coupling under repeated deformation. In the following subsections, we highlight recent representative implementations—ultrathin skin‐conformable electrodes and skin‐interfaced haptic devices—that exemplify how these design criteria can be translated into practical systems for long‐term physiological monitoring and tactile interaction at the skin surface.​

### 4.1 Ultrathin Skin‐Interfaced Electronics

**FIGURE 5:**
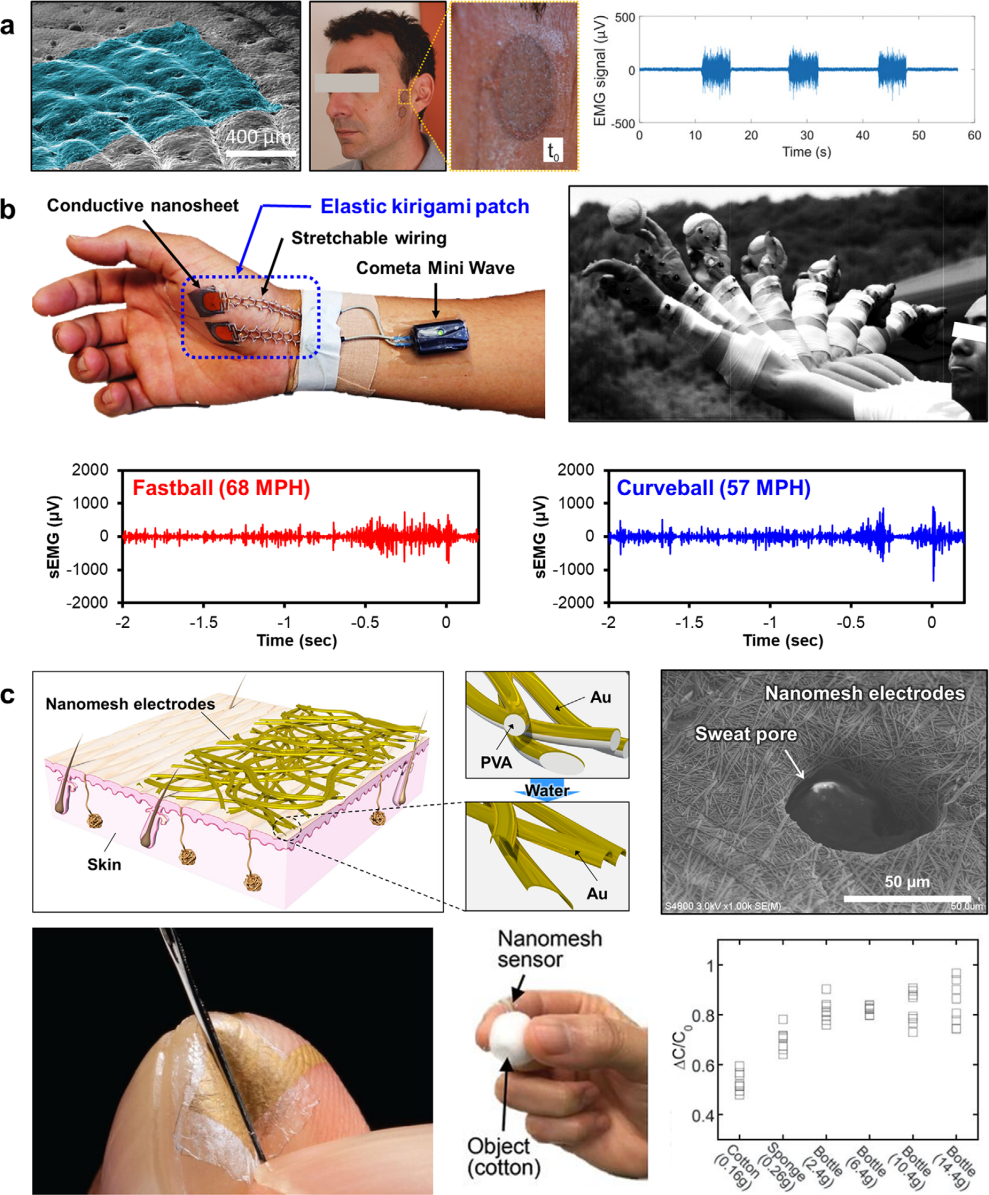
Ultrathin skin‐conformable electrodes. (a) Tattoo electrodes were transferred onto a silicone replica of human skin (colorized SEM micrograph, 45° tilted view) and applied to the mandibular muscle for surface electromyography (sEMG) recording. Reproduced with permission [128]. Copyright 2018, Wiley‐VCH. (b) Elastic kirigami patch composed of conductive nanosheets and elastic kirigami wirings for sEMG recording on the palm during baseball pitching. Top left: photograph and schematic illustration of the patch attached to the skin and connected to a Bluetooth module. Top right: sequential high‐speed images of pitching motion. Bottom: sEMG signals of the abductor pollicis brevis muscle during a 68 MPH fastball throw (left) and a curveball with a speed of 57 MPH (right). Reproduced with permission [7]. Copyright 2019, Springer Nature. (c) On‐skin nanomesh electronics. Top left: schematic illustration of Au nanomesh conductors fabricated by evaporating Au onto electrospun poly(vinyl alcohol) (PVA) nanofibers, followed by water‐assisted PVA removal to achieve conformal adhesion to the skin. Top right: SEM image showing nanomesh electrodes conforming into sweat pores. Reproduced with permission [289]. Copyright 2024, Springer Nature. Bottom left: Nanomesh pressure sensor attached to an index finger. Bottom middle: Grip‐force measurement while grasping a natural object (e.g., cotton ball). Bottom right: Peak grip‐force values for six natural objects; each square denotes one lift. Reproduced with permission [134]. Copyright 2020, AAAS.

Conventional gel electrodes remain the most widely used technology for acquiring electrophysiological signals such as electromyograms (EMG), electroencephalograms (EEG), and electrocardiograms (ECG). However, their long‐term use is often limited by discomfort, dehydration of the gel, and potential skin irritation caused by adhesives. To address these limitations, a wide range of ultrathin, skin‐conformal electrodes that can adhere to the skin without adhesives have been developed and reported in recent years [18, 120, 121, 122, 123, 124, 125, 126, 127, 128, 129, 130, 131, 132]. These devices aim to achieve stable signal acquisition while minimizing mechanical and physiological burden on the skin. Among the various approaches, this section highlights three representative classes—tattoo, nanosheet, and nanomesh electrodes—that exemplify distinct structural and material strategies toward imperceptible, skin‐mounted biosensors (Figure 5).

One representative example is the class of tattoo electrodes (Figure 5a), fabricated by inkjet printing conducting polymers such as poly(3,4‐ethylenedioxythiophene):poly(4‐styrenesulfonate) (PEDOT:PSS) onto commercial temporary tattoo substrates [123, 125, 126, 127, 128, 131, 132]. Their ultrathin morphology and seamless adhesion to human skin enable high‐quality and stable biosignal acquisition in real‐world settings. Multiple studies have demonstrated that tattoo electrodes deliver excellent performance in electrophysiological measurements—including EMG, EEG, and ECG—owing to their low impedance, mechanical transparency, and breathability, which minimizes skin irritation and supports long‐term monitoring. Their breathability and compatibility with skin physiology have been systematically investigated, confirming safe long‐term wear. Clinical validations further showed stable performance in EEG and MEG recordings, benefiting from their all‐polymer, metal‐free structure that eliminates magnetic artifacts. Other reports have detailed nanosheet‐based tattoo electrodes that achieve excellent adhesion and signal quality on skin, while mechanistic studies clarified the capacitive coupling underlying their efficient bio‐interfacing. Collectively, these works establish tattoo electrodes as a versatile and practical platform for noninvasive, wearable biosensing and continuous health monitoring.

Another important category is the ultrathin nanosheet electrode (Figure 5b), which, like tattoo electrodes, utilizes conducting polymers such as PEDOT:PSS but differs in its supporting substrate [7, 121, 122, 133]. Instead of commercial tattoo films, these devices employ polymer nanosheets composed of biodegradable polylactic acid (PLA) [122] or elastomeric styrene–butadiene–styrene (SBS) [7], providing enhanced biocompatibility and versatility. The nanosheet configuration enables direct lamination onto the skin without adhesives, while their total thickness of only a few hundred nanometers ensures mechanical transparency and intimate contact. A further advantage lies in their manufacturability: nanosheets can be fabricated in large areas using scalable roll‐to‐roll processes, establishing a pathway toward cost‐effective mass production [122]. Beyond their mechanical conformity, nanosheet electrodes have been integrated with kirigami‐inspired stretchable interconnects to create skin‐mounted systems capable of stable electrophysiological monitoring even during dynamic motion. This strategy has been successfully demonstrated in sports applications, where palm‐mounted nanosheet patches captured electromyographic signals during baseball pitching without interfering with natural hand movements [7]. Together, these features position nanosheet electrodes as a complementary platform to tattoo‐based systems, offering superior scalability and adaptability for both healthcare and athletic monitoring.

A third approach involves nanomesh electrodes (Figure 5c), fabricated by electrospinning polymer nanofibers such as poly(vinyl alcohol) (PVA) or polyurethane (PU) to form a porous scaffold, followed by thin gold deposition to render conductivity [18, 129, 130, 134, 135]. Their open, gas‐permeable architecture allows natural skin respiration and sweat evaporation, thereby preventing irritation and enabling continuous skin impedance monitoring over extended periods. These properties support long‐term physiological recording with high fidelity, maintaining stable impedance under daily‐life conditions. Beyond impedance sensing, nanomesh electrodes have also been applied as pressure sensors on fingertips [134, 135], where their ultrathin morphology preserves the native sense of touch while accurately detecting tactile forces. Further developments have yielded nanomesh strain sensors with extreme durability against friction and mechanical deformation, enabling reliable signal acquisition even under dynamic and high‐stress conditions. Collectively, these characteristics establish nanomesh electrodes as a versatile and imperceptible platform for bioelectronic applications, uniquely suited for continuous monitoring and tactile sensing.

Despite these advances, several technical challenges remain before ultrathin and breathable electrodes can be fully established as practical standards. Chief among them is ensuring stable electrical and mechanical connections to external wiring, as contact regions often represent the most fragile points both electrically and mechanically. In addition, enhancing water resistance and environmental durability is critical for reliable operation under perspiration and daily‐life conditions. Nevertheless, it is important to emphasize that certain physiological signals can only be captured with such ultrathin, permeable electrodes. Examples include stable impedance monitoring during long‐term wear, high‐precision tactile sensing at the fingertips without interfering with natural perception, and monitoring of fine muscle activities that are difficult to access with conventional gel electrodes. These unique capabilities underscore the indispensable role of ultrathin and breathable electrode platforms in advancing next‐generation wearable and bio‐integrated systems.

### 4.2 Haptic Devices

Haptic technologies represent a rapidly growing frontier in bio‐integrated electronics, aiming to enrich interactions with virtual and augmented reality (VR/AR) systems, rehabilitation tools, and prosthetic feedback by directly engaging the skin's mechanoreceptors [136, 137]. Unlike conventional rigid haptic interfaces, skin‐mounted systems prioritize softness, conformability, and wireless operation, enabling seamless integration with the human body while minimizing discomfort and enhancing user immersion. Recent advances highlight how flexible platforms can deliver programmable tactile sensations in ways that merge the digital and physical worlds [8, 138, 139, 140, 141, 142].

**FIGURE 6:**
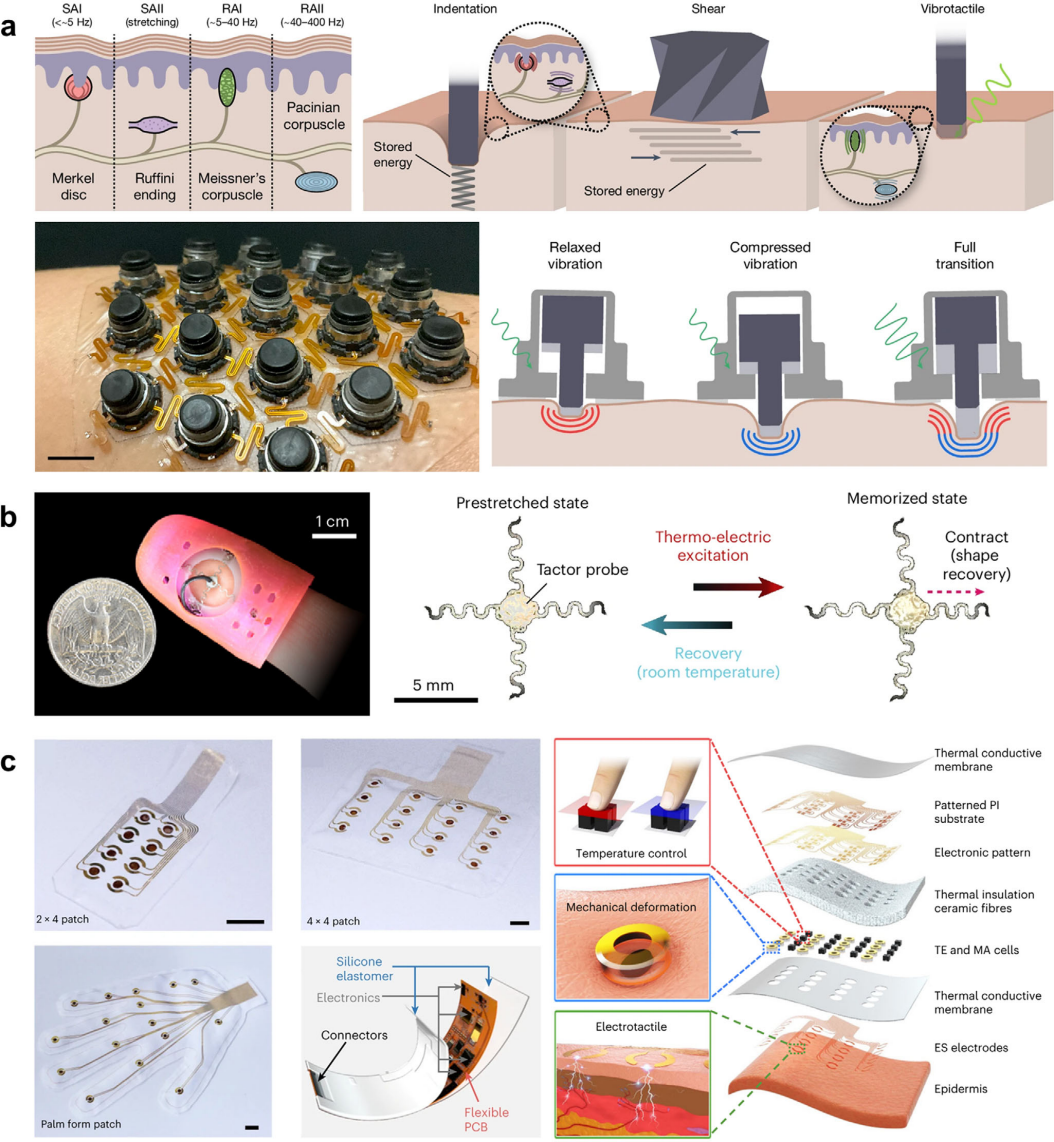
Skin‐interfaced haptic devices. (a) Mechanistic concept of bioelastic state recovery for skin‐interfaced haptic sensory substitution. The skin functions as an elastic energy‐storing medium that enables bistable actuation and efficient delivery of indentation, shear, and vibrotactile stimuli, facilitating targeted activation of cutaneous mechanoreceptors for sensory substitution. Reproduced with permission [138]. Copyright 2024, Springer Nature. (b) Skin‐mounted haptic interface based on serpentine SMA actuators for multimodal cutaneous feedback. Photograph of the finger‐worn device (left) and actuation principle (right). Four serpentine SMA actuators, arranged in antagonistic pairs, are prestretched and subsequently contracted by Joule heating, producing controlled motion of a central tactor to deliver normal and tangential cutaneous cues. State recovery after stimulation is assisted by cooling of the SMA and the elastic restoring force of fingertip skin, enabling repeatable tactile patterns. Reproduced with permission [139]. Copyright 2025, Springer Nature. c) Multimodal haptic feedback system. Photographs of haptic feedback arrays with different layouts (2 × 4 patch, 4 × 4 patch, and palm‐form patch) and a schematic illustration of the wireless control circuit based on a flexible printed circuit board. Exploded‐view diagram of a single device comprising sixteen independently addressable multimodal haptic units, each integrating one mechanical actuator (MA), two pairs of thermoelectric (TE) pellets for thermal cues, and one pair of electrotactile (ES) electrodes for electrical stimulation. PI: polyimide. Reproduced with permission [140]. Copyright 2023, Springer Nature.

As illustrated in Figure 6a, the concept of bioelastic state recovery for haptic sensory substitution leverages the intrinsic elasticity of human skin as an active mechanical element in signal transduction [138]. In this approach, the skin is not treated as a passive support but rather as an energy‐storing medium that deforms elastically under actuation and subsequently recovers its original state. Soft haptic transducers apply controlled normal or shear deformations to the skin, temporarily storing mechanical energy within the elastic layers of the epidermis and dermis. Upon release or transition of the actuator state, this stored elastic energy drives a rapid and reversible recovery of skin deformation, generating distinct spatiotemporal mechanical stimuli that effectively engage both rapidly and slowly adapting mechanoreceptors. By exploiting the natural elasticity and viscoelastic recovery of skin, this bioelastic mechanism enables bistable operation, low‐energy actuation, and repeatable tactile feedback without continuous power input. Such skin‐integrated haptic systems demonstrate an effective pathway for sensory substitution, converting non‐tactile sensory information into intuitive and perceivable cutaneous cues, with strong potential for immersive interfaces and medical rehabilitation applications.

A second example, shown in Figure 6b, presents a flexible skin‐mounted haptic interface for multimodal cutaneous feedback [139]. This lightweight, finger‐worn platform is built on serpentine shape‐memory alloy actuators and can reproduce eleven distinct tactile motions—including lateral, diagonal, rotational, and normal forces—thereby delivering nuanced cutaneous sensations. Its soft 3D‐printed architecture ensures conformability and comfort while enabling users to interact seamlessly with both virtual and real‐world environments. Importantly, the device achieves multidirectional actuation without bulky hardware, offering a promising route toward lifelike VR/AR experiences and assistive technologies.

As shown in Figure 6c, a skin‐integrated multimodal haptic interface for immersive tactile feedback demonstrates a soft, wireless, and battery‐free platform capable of multimodal stimulation, including vibration, skin stretch, and thermal cues [140]. Designed as a thin, conformal sheet laminated directly onto the skin, the system integrates arrays of programmable actuators to deliver spatiotemporally resolved feedback across large body areas. Applications span VR/AR immersion, prosthetic feedback, and social communication, marking a milestone in the integration of haptic sensations into skin electronics.

In summary, skin‐interfaced haptic devices are transitioning from bulky prototypes to soft, conformal systems that can reproduce diverse tactile modalities with high fidelity. The demand for such technologies is particularly high in the context of human–machine interfaces, where naturalistic feedback and seamless integration with the body are essential. Key challenges remain in ensuring reliable power delivery, scalable integration of actuator arrays, and long‐term skin safety. At the same time, achieving precise tactile interaction, especially at sensitive sites such as the fingertips, will benefit greatly from the ultraconformable electrodes and sensors introduced in the previous section. These capabilities open the door to a wide range of applications, from immersive VR/AR and motion augmentation to robotic hands requiring fine tactile perception. Beyond these, medical scenarios represent another critical frontier: haptic interfaces can enhance prosthetic limb control [143, 144], support motor rehabilitation therapies [145, 146, 147], and provide surgeons with tactile feedback in robotic‐assisted procedures [148, 149, 150]. Collectively, these advances underscore the transformative potential of skin‐mounted haptics in shaping the future of both daily human–machine interactions and clinical practice.

## 5 Neural Interfaces

**FIGURE 7:**
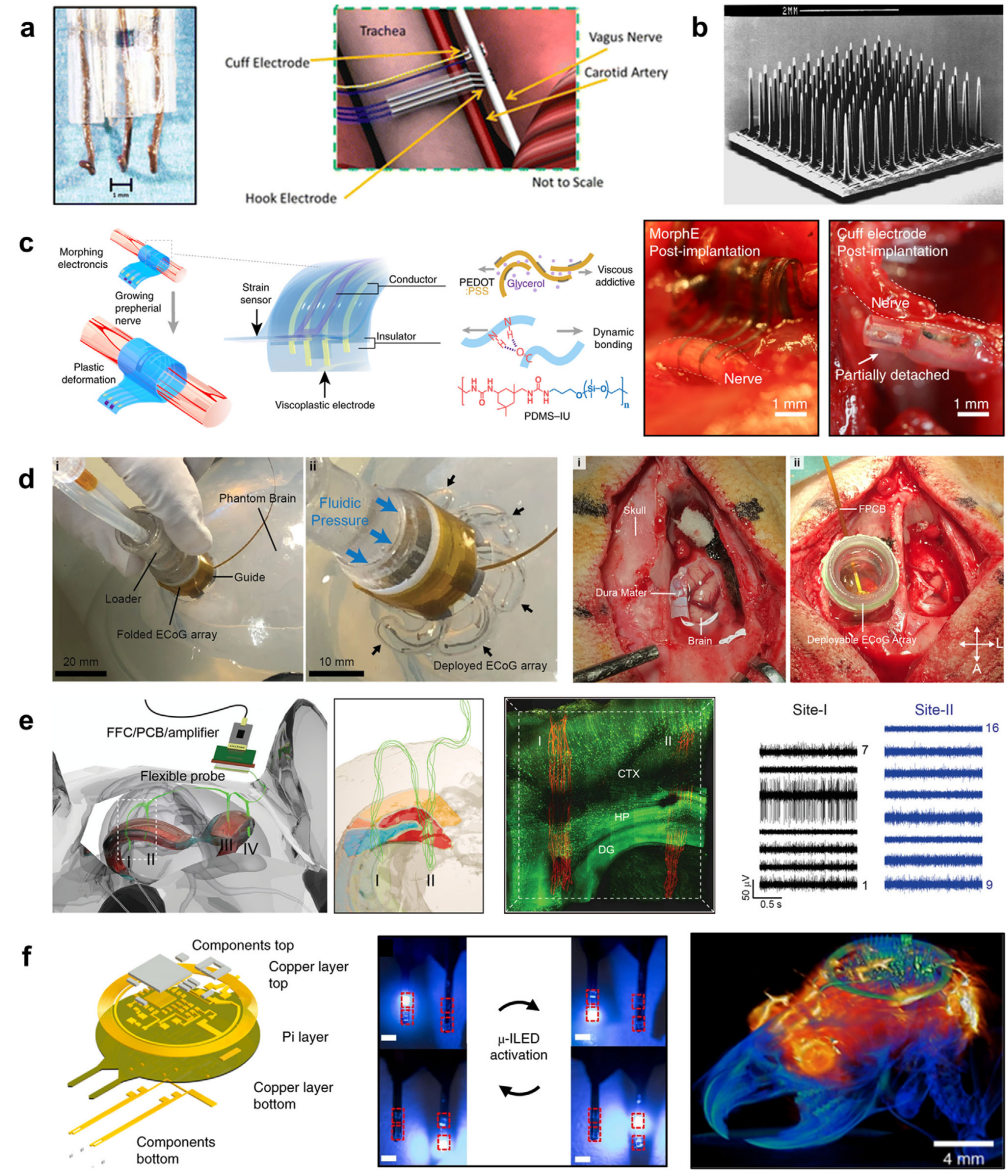
Neural interfaces. (a) Electrophysiological recording system for the cervical vagus nerve. A hook electrode with three leads was used to record cervical vagus nerve activity. An artist rendering shows the isolated cervical vagus nerve and the placement of the hook and cuff electrodes. Reproduced with permission [151]. Copyright 2018, Springer Nature. (b) Utah Intracortical Electrode Array. The structure measures 4.2 mm on a side, and each electrode is 1.2 mm in length. Reproduced with permission [154]. Copyright 1997, Elsevier Inc. (c) MorphE based on viscoplastic electronic materials that conformally adapt to sciatic nerve growth during rat adolescence. The device combines PEDOT:PSS plasticized with glycerol as a soft conductor and a PDMS–IU/MPU blend as a viscoplastic insulator, enabling plastic deformation under nerve elongation. After four weeks, MorphE remained attached to the nerve, while a conventional cuff electrode detached. Scale bar, 1 mm. Reproduced with permission [156]. Copyright 2020, Springer Nature. (d) Deployment of a soft robotic ECoG system. Left: Deployment of a folded ECoG array through a burr hole in a phantom brain model and fluidic‐pressure‐driven expansion of the electrode array. Right: Surgical deployment and conformal placement of the ECoG array on the cortical surface. Reproduced with permission [159]. Copyright 2023, AAAS. (e) Stitching flexible electronics into the brain using a glass needle. Left: Schematic of multi‐site (I–IV) implantation of a single flexible neural probe across the mouse brain. Middle: Three‐dimensional visualization of probe–neuron interfaces in a YFP‐H mouse brain two weeks post‐implantation (scale bar, 400 µm). Right: Representative multi‐channel neural recordings from different implantation sites. Reproduced with permission [162]. Copyright 2023, Wiley‐VCH. (f) Fully implantable, battery‐free optoelectronic system integrating four µ‐ILEDs for bilateral, multimodal neural modulation. Left: Exploded‐view schematic of a fully implantable, battery‐free optoelectronic system showing multilayer construction (components, copper layers, and polyimide (PI) substrate) and bilateral injectable probes integrating four µ‐ILEDs for neural modulation. Middle: Demonstration of a bilateral multi µ‐ILED device with sequential activation of all stimulating µ‐ILEDs (scale bars: 0.5 mm). Right: Magnetic resonance imaging–computed tomography (MRI–CT) composite rendering showing the implanted bilateral device. Reproduced with permission [175]. Copyright 2018, Springer Nature.

The ability to monitor and modulate neural activity with high precision is a central goal in bioelectronics. While skin‐mounted sensors provide valuable insights, the information obtainable from the body surface remains inherently limited, making it a natural step to directly interface electronics with peripheral and central nerves. Traditional neural electrodes have historically been developed along two major directions, reflecting the anatomical and functional differences between the peripheral and central nervous systems. Conventional approaches include cuff‐ or hook‐shaped electrodes designed to wrap around peripheral nerves [151, 152, 153] (Figure 7a), as well as penetrating needle‐type electrodes primarily used for intracortical recordings in the brain [154, 155] (Figure 7b). While these devices have enabled fundamental advances in neural recording and stimulation, their largely rigid or semi‐rigid geometries are poorly matched to the soft, viscoelastic, and dynamically evolving nature of neural tissues. This mismatch often leads to mechanical irritation, foreign‐body responses, and long‐term signal degradation. In response, recent developments in neural interfaces increasingly focus on tailoring device architectures, materials, and mechanics to the specific structural and mechanical properties of the target tissue. In the following sections, we highlight representative examples of such tissue‐adaptive designs for peripheral nerves and the brain, respectively.

### 5.1 Peripheral Nerve Interfaces

Peripheral nerve interfaces must accommodate large, repetitive deformations as well as long‐term changes in nerve geometry associated with growth, regeneration, and physiological motion. Conventional cuff‐ or hook‐type electrodes with fixed geometries are poorly suited for such dynamic environments, often leading to mechanical irritation, chronic inflammation, and signal instability over time. Morphing neural interfaces exemplify this new direction. These devices employ soft, viscoplastic electronic materials that undergo permanent deformation only under very slow strain rates, enabling gradual adaptation to nerve growth (Figure 7c) [156]. The morphing electronics (MorphE) device shows essentially zero residual stress at growth‐like strain rates of ∼10^−^^5^–10^−^^4^ %/s, yet sustains elastic behavior with a modulus of ∼0.4 MPa at fast strains of 50%/s. Such growth‐adaptive morphing maintains conformal contact as peripheral nerves enlarge in diameter (up to ∼2.4‐fold over two months in rapidly growing rats) while minimizing chronic inflammation and preserving stable stimulation and recording—outcomes that are difficult to achieve with conventional cuff or hook electrodes of fixed geometry. At the same time, these systems highlight the dual role of viscoelasticity and dynamic bonding chemistry: they are actively exploited to enable gradual shape adaptation and self‐healing assembly during surgery, yet their time‐dependent stress relaxation and interfacial rearrangement also dictate how stresses are redistributed at the tissue–device interface, thereby constraining long‐term interface design.

### 5.2 Brain Interfaces

In contrast to peripheral nerves, interfaces targeting the brain face a distinct set of challenges arising from the extreme softness of neural tissue, confined surgical access, and stringent requirements for long‐term biocompatibility. Conventional electrocorticography (ECoG) grids require large craniotomies to expose the cortical surface for placement, which increases surgical invasiveness and limits coverage area [157, 158]. To address this, deployable ECoG systems (Figure 7d) have been developed that can be inserted through a small burr hole and then unfold autonomously beneath the dura [159]. Using soft robotic actuation and pressure‐driven eversion mechanisms, these prefolded arrays expand to cover wide cortical regions without requiring direct manual manipulation. Once deployed, they conform closely to the brain surface, achieving high‐resolution, large‐area recordings while reducing trauma. This self‐deployable strategy represents a paradigm shift in how cortical interfaces can be delivered and positioned, paving the way for safer and less invasive neurosurgical procedures.

To further minimize mechanical mismatch with brain tissue, next‐generation intracortical and subdural probes are being engineered to exhibit “tissue‐like” mechanical properties [16, 160, 161, 162, 163, 164, 165]. Conventional penetrating electrodes, though capable of high spatial resolution, often trigger chronic inflammation and glial scarring due to mechanical mismatch with soft neural tissue, leading to gradual signal loss. To overcome this limitation, mesh electronics composed of submicrometer‐thick conductive traces embedded in open polymer networks have been developed, exhibiting flexural rigidity comparable to brain tissue (10^−4^–10^−1^ nN m) [16]. These structures can be injected through fine needles, where they unfold and integrate seamlessly with minimal immune response, enabling stable electrical recordings over several months. Extending this concept, stitched flexible electronics use thin, cable‐like probes that can be precisely “sewn” into 3D brain structures, creating dense recording networks across cortical and subcortical regions while minimizing insertion trauma (Figure 7e) [162]. Together, these strategies establish a new paradigm for deep‐brain interfacing—where electronics no longer behave as foreign objects but rather as tissue‐like components—laying the groundwork for chronic, high‐fidelity communication between neural circuits and artificial systems.

Optogenetics—an approach that combines optical and genetic techniques to control the activity of specific neurons with light—has revolutionized neuroscience by enabling precise spatiotemporal modulation of neural circuits [166, 167, 168, 169, 170, 171, 172, 173, 174]. Deep‐brain regions, meanwhile, present an additional level of complexity. Accessing and modulating neuronal activity at depth requires devices that are both miniaturized and capable of wireless operation, as tethered systems restrict movement and cause tissue damage. Fully implantable optoelectronic platforms (Figure 7f) meet this need by integrating microscale inorganic LEDs at the tips of injectable probes [169, 175]. These probes deliver localized optogenetic stimulation deep within the brain without the need for external power cables or bulky batteries. Their wireless, battery‐free architecture enables long‐term studies in freely moving animals, providing powerful tools for dissecting and modulating neural networks with high spatiotemporal precision.

Together, these advances demonstrate how innovations in structural mechanics—such as morphing, deployable, and mesh architectures exemplified by fiber‐like mesh electrodes—and in materials science—particularly ultrathin, highly compliant conductor networks—are transforming the design philosophy of neural interfaces. For peripheral nerve systems, soft and adhesive electrodes that can maintain stable contact under large and repetitive deformations are essential to ensure reliable signal coupling during continuous body motion. For cortical ECoG interfaces, reducing film thickness and enhancing adhesion minimizes surgical invasiveness while enabling conformal, large‐area neural mapping. For deep‐brain implants, achieving ultralow mechanical stiffness is crucial to avoid chronic tissue damage and sustain long‐term integration within fragile neural structures. Across these distinct modalities, the unifying goal is to achieve mechanical and biological harmony between electronic and neural systems. Such progress opens new clinical frontiers—from restoring motor and sensory function to treating neurological disorders such as epilepsy [157, 176], Parkinson's disease [177, 178], and Alzheimer's disease [179, 180]—while future integration with soft robotics, bioresorbable materials, and wireless power systems will enable the next generation of intelligent, minimally invasive neural implants.

## 6 Dynamic Wet‐Organ‐Adhesive Electronics

Interfacing bioelectronic devices with internal organs presents challenges that are fundamentally distinct from those encountered at the skin surface or in neural tissues. Compared with the brain or peripheral nerves, which remain relatively static and are therefore more accessible for stable device attachment, many internal organs undergo vigorous and continuous motion due to peristalsis, respiration, or cardiac cycles. Moreover, their surfaces are typically covered with moist, slippery layers such as serosa or mucosa, making adhesion substantially more difficult than on the relatively dry epidermis. Traditional fixation methods have relied on suturing, but these approaches can cause additional tissue damage, trigger inflammatory responses, and fail to provide long‐term mechanical stability. To address these issues, recent research has focused on developing bioadhesive and tissue‐adhesive materials that enable conformal, suture‐free, and durable integration of electronic systems with wet and dynamic organ surfaces [181, 182, 183, 184, 185, 186, 187, 188, 189, 190, 191, 192, 193].

**FIGURE 8:**
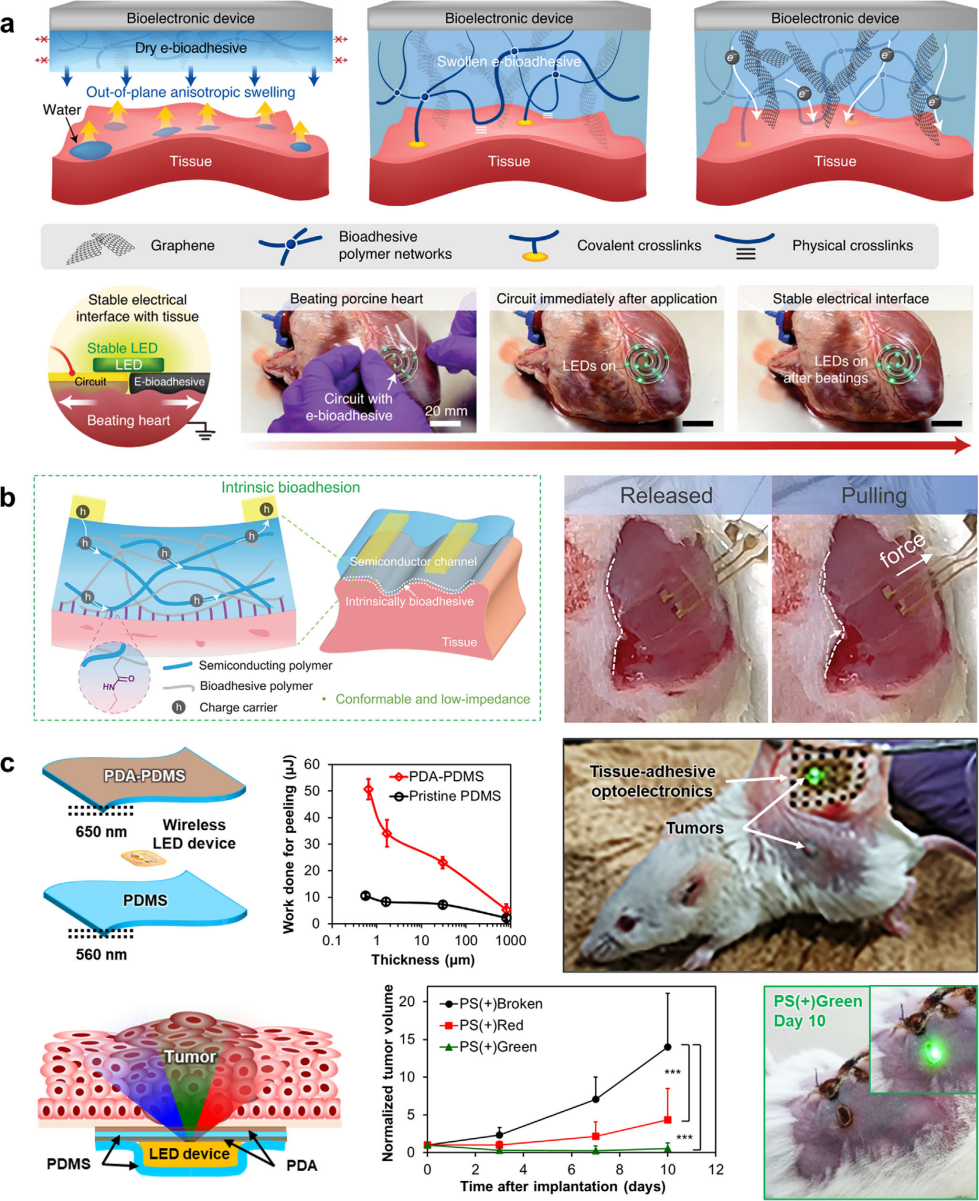
Tissue‐adhesive electronics. (a) Electrical bioadhesive interface for bioelectronics. Top: graphene nanocomposite‐based e‐bioadhesive design exhibiting anisotropic out‐of‐plane swelling, rapid and robust adhesion to wet tissues, and high electrical conductivity. Bottom: schematic and photographs showing stable LED operation of a circuit adhered to an ex vivo porcine heart using the e‐bioadhesive interface. Reproduced with permission [189]. Copyright 2021, Springer Nature. (b) Bioadhesive polymer semiconductors for tissue‐interfaced electrochemical transistors. Left: Schematic of a bioadhesive semiconductor forming covalent bonding with wet tissue via an integrated semiconducting–adhesive polymer network. Right: Photographs showing stable adhesion of the device on gastrocnemius muscle under mechanical pulling. Reproduced with permission [190]. Copyright 2023, AAAS. (c) Tissue‐adhesive optoelectronics for implantable, wirelessly powered metronomic photodynamic therapy (mPDT). Top left: construction of a tissue‐adhesive LED device with an NFC chip encapsulated between PDA–PDMS and pristine PDMS nanosheets; plot shows adhesion energy versus film thickness. Right: implantation image of the device on the inner dorsal skin of a mouse. Bottom: schematic of localized photoirradiation and tumour growth curves comparing control and PDT groups (*n* = 10). Inset: wirelessly lit LED beneath skin on day 10. Reproduced with permission [183]. Copyright 2019, Springer Nature.

One strategy employs electrical bioadhesive platforms that integrate polymer networks capable of forming covalent and physical crosslinks with hydrated tissues (Figure 8a) [189]. Upon application, the bioadhesive swells anisotropically, filling interfacial gaps and establishing robust bonding. This property allows electronic circuits encapsulated within the adhesive to maintain stable operation on beating porcine hearts, including continuous LED illumination without detachment or electrical failure. By integrating chemical adhesion into the device architecture, such systems overcome the limitations of sutures and ensure reliable electrical contact even under intense mechanical perturbations.

Another direction involves bioadhesive polymer semiconductors, in which intrinsic semiconducting and bioadhesive functionalities are combined within a single conjugated material (Figure 8b) [190]. Functional side groups along the polymer backbone form stable covalent bonds with tissues, achieving both electrical conductivity and strong adhesion. These materials enable organic electrochemical transistors (OECTs) that conform to dynamically moving organs while maintaining low interfacial impedance. Demonstrations on living cardiac and skeletal muscle tissues confirmed stable electrophysiological recording and stimulation under continuous motion, showing how molecular‐level bioadhesion can simplify device design and enhance chronic stability.

A third approach explores tissue‐adhesive optoelectronic systems for localized cancer therapy (Figure 8c) [183]. In this design, ultrathin PDMS nanosheets coated with polydopamine (PDA)—a mussel‐inspired bioadhesive polymer [194, 195]—encapsulate wireless LED chips, achieving direct, suture‐free attachment to moist organ surfaces. The ultrathin geometry ensures conformability and low flexural rigidity, while PDA chemistry provides durable bonding in wet environments. In vivo experiments demonstrated wireless, long‐term localized illumination for tumor treatment in animal models, enabling metronomic photodynamic therapy (mPDT) [183, 196, 197, 198, 199]. This concept illustrates how combining nanosheet mechanics with bioinspired chemistry opens new possibilities for minimally invasive, tissue‐adhesive optoelectronics in oncology and regenerative medicine.

Achieving stable and long‐term adhesion of electronic devices to internal, wet organs is far more challenging than integration with the skin. Highly dynamic organs such as the heart and gastrointestinal tract constantly deform, while fragile tissues like the brain cannot be sutured, requiring devices that conform intimately without mechanical stress. In such environments, chemical adhesion via covalent bonding—using materials like polydopamine or hydrogels—is indispensable, as van der Waals forces alone cannot ensure durability. Moreover, maintaining a stable interface demands precise control of interfacial hydration and ionic conductivity to accommodate fluid exchange and mechanical motion. These principles form the foundation of “electronics that become part of the tissue,” emphasizing that reliable bio‐integration requires not only mechanical compliance but also chemical affinity with living matter. To achieve true seamless integration with biological tissues, however, the electronic materials themselves must be made as soft and deformable as the tissues they interface with—an objective that drives the emerging development of liquid metal‐based conductors introduced in the following section.

## 7 Next‐Generation Materials for Bio‐Integrated Electronics

In Sections 2, 3, 4, 5, 6, we have focused primarily on how to engineer mechanical compatibility between electronic systems and diverse organs—starting from fundamental tissue mechanics, then introducing general design principles, and finally demonstrating tissue‐specific platforms for the skin, nervous system, and dynamic wet organs. These sections establish that appropriate combinations of ultrathin substrates, stretchable geometries, and bioadhesive interfaces can substantially reduce mechanical mismatch and enable stable, long‐term integration on or within living tissues. Building on this foundation, this section shifts the perspective from structural design to materials choice, highlighting emerging classes of conductors and substrates that provide functionalities unattainable with conventional soft electronics. In particular, we first examine liquid metal–based systems that couple metallic‐level conductivity with fluid‐like deformability for wearable and implantable devices, and then discuss biodegradable transient materials that introduce time‐programmed disappearance and resorption. These next‐generation materials, when combined with the design strategies outlined in the earlier sections, open pathways toward biointegrated electronic platforms that are not only mechanically matched to target tissues but also reconfigurable or vanishing in time.

### 7.1 Liquid Metal‐Based Soft Electronics for Wearable and Implantable Devices

A critical step toward stable and long‐term integration of bioelectronic devices is ensuring robust adhesion to biological tissues, as described in the previous section. Once adhesion is secured, the next challenge is to minimize the mechanical stress imparted to the tissue by the electronic system. Ideally, wiring and interconnects should be as soft and deformable as the surrounding biological structures, thereby avoiding irritation or functional interference. In this sense, the most desirable configuration is analogous to human vasculature, in which compliant vessels serve as soft channels for fluid transport. Similarly, creating “fluidic interconnects” on tissue surfaces offers a pathway to achieving both mechanical imperceptibility and stable electrical performance. This perspective naturally highlights liquid metals as ultimate candidates for constructing soft wiring in tissue‐integrated electronics.

**FIGURE 9:**
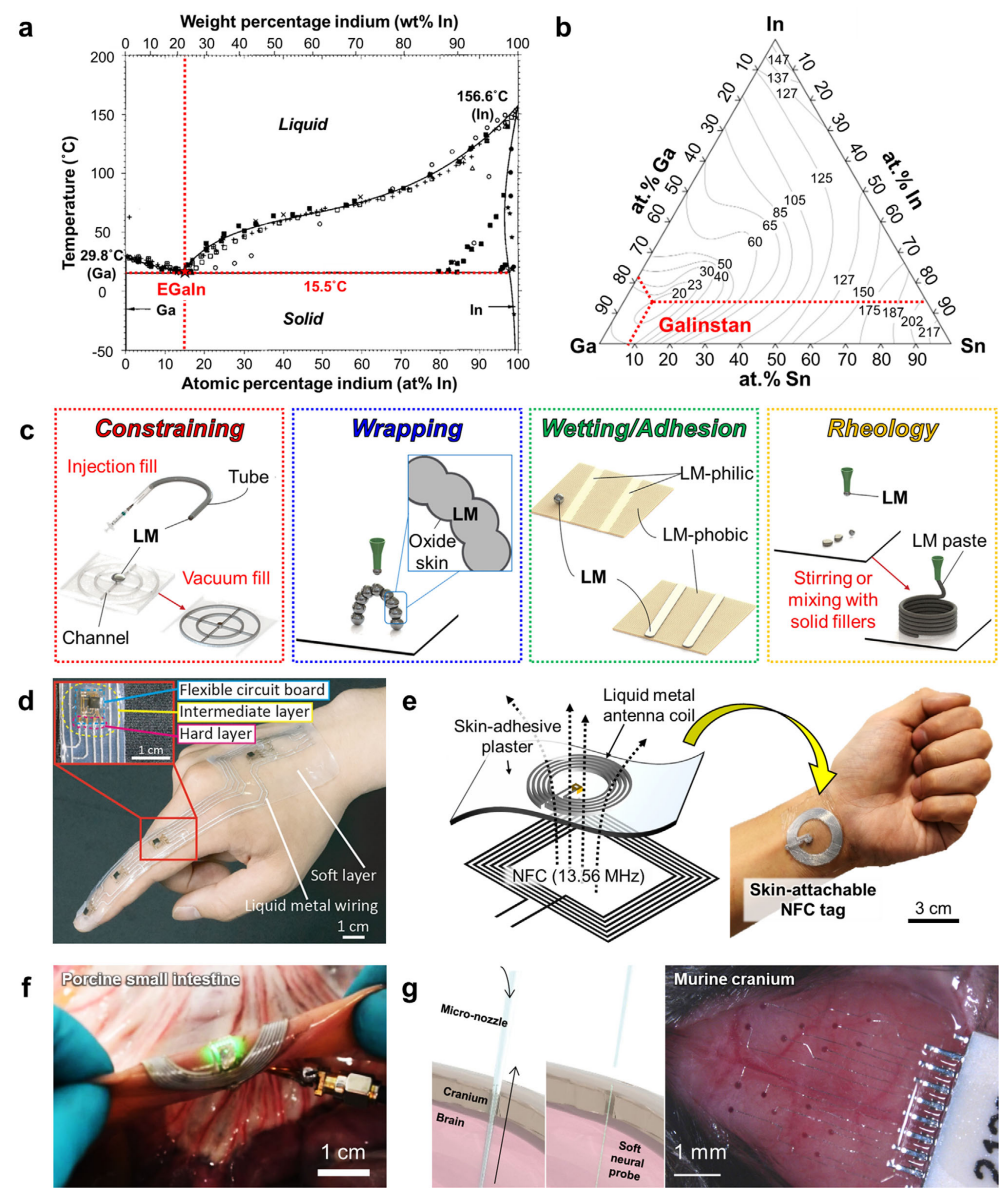
Liquid metal‐based soft electronics. (a) Ga–In binary phase diagram showing the eutectic composition and melting point near 15.5°C, as verified by experimental data. Reproduced with permission [206]. Copyright 2007, Springer Nature. (b) Liquidus projection of the Ga–In–Sn ternary phase diagram highlighting the compositional region corresponding to Galinstan. Contours indicate liquidus temperatures (°C) as a function of Ga, In, and Sn atomic fractions, as compiled from the Linus Pauling File (SpringerMaterials) database, which experimentally maps phase fields and crystalline compounds for inorganic Ga–In–Sn alloys. Reproduced with permission [210]. Copyright 2016, Springer & Material Phases Data System (MPDS). (c) General strategies for controlling liquid shape, including for liquid metals (LM): constraining, wrapping, wetting/adhesion, and rheology modification. Reproduced with permission [214]. Copyright 2023, Wiley‐VCH. (d) Soft intelligent systems integrating stretchable hybrid devices with machine learning. Finger‐mounted device with four IMUs connected by LM wires for motion recognition. Reproduced with permission [255]. Copyright 2024, Elsevier Inc. (e) Schematic illustration and photograph of a skin‐attachable NFC tag that uses a liquid‐metal antenna coil patterned directly onto a commercially available skin‐adhesive plaster for conformal wireless operation on the wrist. Reproduced with permission [119]. Copyright 2024, Wiley‐VCH. (f) Ultra‐deformable, tissue‐adhesive liquid metal antenna with high wireless powering efficiency. Left: free‐standing light‐emitting device with embedded LEDs activated by wireless power during deformation. Right: antenna attached to porcine intestine during stretching and compression, maintaining wireless operation. Reproduced with permission [118]. Copyright 2021, Wiley‐VCH. (g) Schematic illustration of syringe‐based injection of a soft neural probe, in which the probe is printed and delivered through a micro‑nozzle by balancing the injection flow rate and nozzle withdrawal velocity. Optical stereomicrograph of a liquid‑metal–based neural interface conformally applied to the surface of a murine cranium. Reproduced with permission [232]. Copyright 2024, AAAS.

As introduced in Section 3, liquid metals represent an exceptionally attractive class of materials for constructing stretchable wiring and circuits [200]. Gallium‐based alloys in particular are promising due to their low toxicity, high electrical conductivity, and chemical stability, making them suitable for a broad range of wearable and implantable applications [34, 201, 202, 203, 204, 205]. Their fundamental characteristics are illustrated in Figure 9, which summarizes their composition, processing methods, and representative device demonstrations.

Gallium‐based alloys such as eutectic gallium–indium (EGaIn) [33, 206, 207, 208] and gallium–indium–tin (Galinstan) [209, 210, 211] remain liquid at or below room temperature. These eutectic alloys possess melting points significantly lower than those of their constituent elements, exhibiting mercury‐like fluidity without its toxicity [212]. Their metallic‐level conductivity (∼10^6^ S/m) combined with fluidic deformability makes them uniquely suited for mechanically adaptive electronics [34, 201, 213]. Adjusting the mixing ratios of constituent metals can further modulate the melting point and phase stability, allowing operation to be tailored to specific environments (Figure 9a,b) [206, 210].

Patterning techniques for liquid metals have evolved rapidly, reflecting advances in additive manufacturing and an improved understanding of interfacial behavior (Figure 9c) [214, 215]. Methods such as injection or vacuum filling into microchannels and hollow fibers enable precise confinement of liquid metals within defined geometries, forming reliable conductive pathways while preventing leakage [118, 119, 216, 217, 218]. Direct writing and extrusion printing approaches utilize the naturally formed oxide skin of gallium‐based alloys, which imparts sufficient yield stress and viscoelasticity to maintain filamentary shapes during printing, effectively allowing a liquid to behave as a printable medium while retaining metallic conductivity [219, 220, 221, 222, 223, 224, 225, 226, 227, 228, 229, 230, 231, 232]. In contrast, selective wetting and adhesion patterning require effective removal of the oxide layer to control surface energy and achieve clean deposition on liquid‐philic regions while suppressing spreading on liquid‐phobic areas [204, 233, 234, 235, 236, 237, 238]. By finely tuning this oxidation–reduction balance at the metal–substrate interface, high‐resolution, site‐specific patterning can be achieved without physical masks [236, 239, 240, 241, 242]. In addition, transfer printing and rheological modification techniques, including the formulation of liquid metal pastes and composites, further enhance processability, scalability, and interfacial adhesion to soft substrates. By simply stirring or mechanically mixing gallium‐based liquids with solid fillers, foams, and liquid–solid mixtures with tunable density, thermal conductivity, and viscoelasticity can be obtained, which are readily extruded or printed onto 3D surfaces [114, 115, 243, 244, 245, 246, 247, 248, 249, 250, 251, 252, 253]. Together, these approaches establish a versatile toolbox for additive, mask‐free fabrication of reconfigurable liquid metal electronic systems with controlled geometry, adhesion, and conductivity.

Recent demonstrations have applied these principles to create highly deformable wearable devices [119, 203, 254]. For example, wearable glove‐type systems incorporating liquid metal interconnects in an island–bridge layout can recognize hand gestures in real time when combined with machine learning algorithms (Figure 9d) [255]. The circuits maintain stable conductivity even under 100% strain, illustrating the robustness of liquid metal wiring in dynamic human–machine interfaces. Building on this concept, direct ink writing (DIW) has been extended to fully 3D‐printed, multilayered microfluidic architectures that embed liquid metal conductors [36]. By formulating a fast‐curing silicone sealant and optimizing the printing conditions, self‐supporting hollow channels with vertical interconnects can be printed without sacrificial supports, enabling stretchable double‐layer microchannels that can be filled with aqueous solutions or liquid metals without collapse or leakage. Using this strategy, a skin‐attachable near‐field communication (NFC) tag was fabricated in which a liquid metal microfluidic antenna coil and an embedded integrated circuit (IC) chip were printed onto a medical‐grade, elastic skin adhesive plaster; the resulting device exhibited a resonance frequency around 14 MHz with a high Q factor and conformed tightly to curved regions such as the wrist while allowing wireless data readout via a smartphone (Figure 9e) [119].

Another major application is the development of liquid metal‐based antennas fabricated on ultrathin elastomeric substrates (Figure 9f) [118]. Using DIW‐printed silicone microchannels [36] infused with Galinstan, antenna coils were fabricated on ultrathin elastomeric substrate as thin as 7 µm, maintaining wireless power transfer efficiency under bending, stretching, and twisting. Coating the devices with polydopamine further enables suture‐free adhesion to wet, moving tissues such as the intestine, where conventional rigid antennas fail to conform. These antennas have successfully powered microscale LEDs via NFC coupling, demonstrating their potential for minimally invasive wireless implants. Expanding beyond single‐layer structures, multilayered and vertically interconnected liquid metal microfluidic circuits have been integrated with functional IC chips to realize skin‐conformal NFC tags, extending applications to wearable health monitoring [119].

Liquid metal wiring has also been extended from skin‐mounted and visceral devices to neural interfaces on the cranium [230, 232, 256]. In one approach, high‐resolution liquid metal “neuro‐electronics” are directly printed onto the skull to form soft interconnects that route signals from implanted flexible neural probes. By tuning the dispensing conditions, the micro‐nozzle can inject liquid metal–filled soft probes through small cranial openings while simultaneously laying down planar interconnects on the bone surface, enabling minimally invasive integration of deep‐brain electrodes with conformal, low‐modulus wiring that reduces mechanical mismatch at the brain–skull interface (Figure 9g) [232].

In summary, liquid metal‐based soft electronics provide a uniquely versatile platform for constructing highly deformable, electrically conductive, and biocompatible systems. Remaining challenges include secure encapsulation to avoid metal leakage, preservation of electrical and mechanical stability over chronic operation in vivo, and establishment of protocols for safe handling and removal in the event of device failure or accidental exposure. Paste‐like or composite formulations that immobilize liquid metals within elastomeric or particulate matrices may offer a practical compromise between safety, printability, and mechanical compliance for clinical translation. Even so, the combination of metallic‐level conductivity with fluid‐like mechanics firmly positions gallium‐based liquid metals as key enabling materials for the next generation of wearable and implantable bioelectronic systems.

### 7.2 Transient Electronics Using Bio‐Degradable Materials

The ultimate goal of implantable bioelectronic systems is to provide therapeutic or diagnostic functions without leaving a permanent foreign body inside the patient. Conventional devices often require surgical removal once their role has been fulfilled, adding additional risks and costs. In contrast, transient electronics are designed to chemically degrade into smaller molecular fragments and subsequently disappear physically after a defined operational period, through processes such as dissolution, resorption, or biodegradation within the body [257]. This concept offers unique advantages for temporary implants, such as post‐operative monitoring, localized therapy, or short‐term stimulation, where long‐term presence is unnecessary or even detrimental. Progress in this field has been driven by the development of biodegradable metals, polymers, and elastomers that combine functional performance with predictable degradation behavior.

**FIGURE 10:**
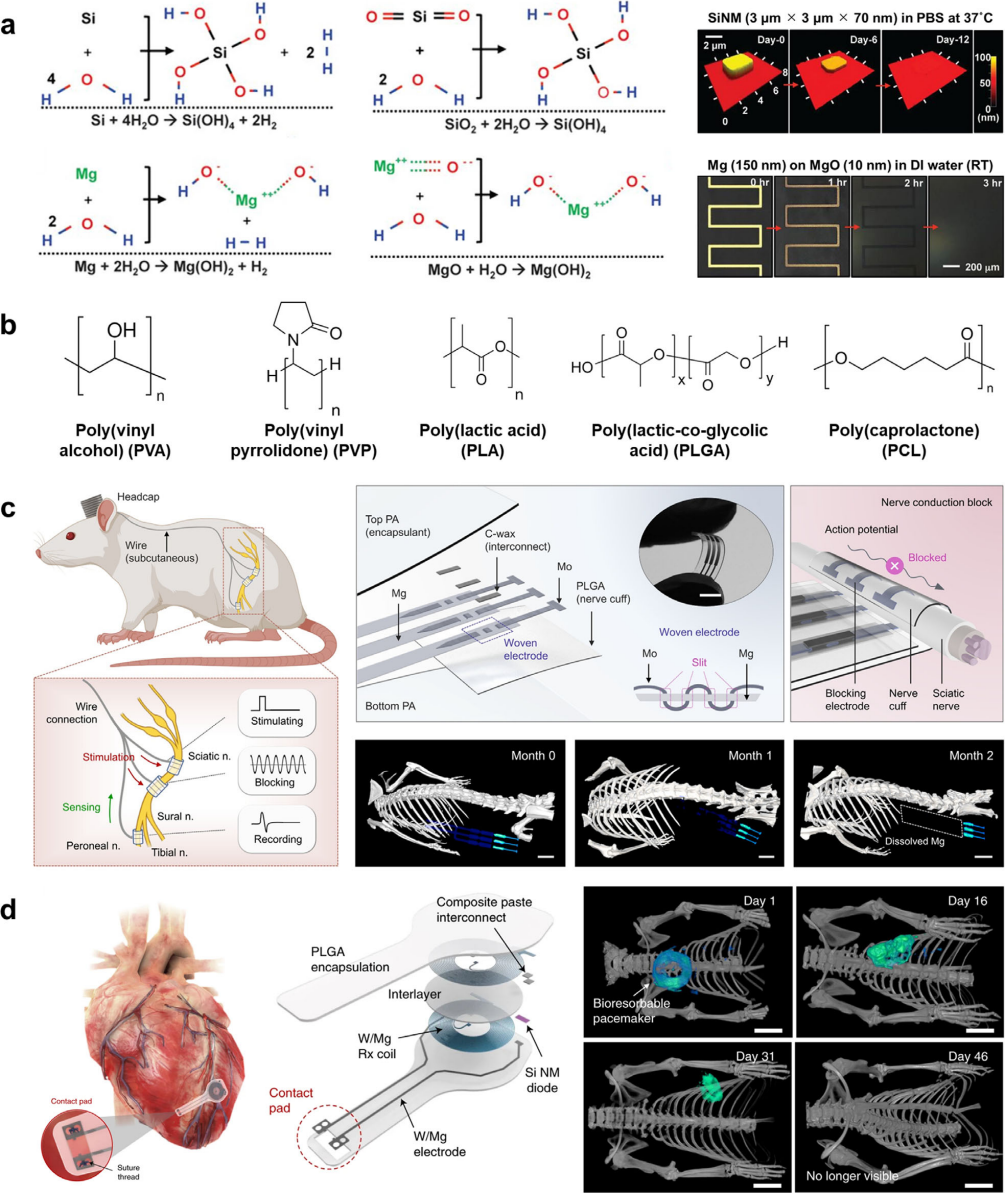
Transient electronics. (a) Hydrolysis reactions of constituent materials (Si, Mg, MgO) with water. Atomic force microscope images show progressive hydrolysis of a Si nanomembrane (3 mm × 3 mm × 70 nm) in PBS at 37°C, and optical microscope images show dissolution of a Mg serpentine trace (150 nm thick) on MgO (10 nm thick) in DI water at room temperature. Reproduced with permission [258]. Copyright 2012, AAAS. (b) Representative chemical structures of biodegradable polymers used in transient electronic systems. (c) Bioresorbable peripheral nerve stimulator for electronic pain block. Left: schematic of device implantation showing subcutaneous routing and nerve cuffs for stimulation, blocking, and recording. Right top: exploded view and photograph of the woven‐interconnect stimulator encapsulated in bioresorbable polymer layers, with exposed Mo electrodes for nerve stimulation and conduction block. Right bottom: 3D CT images of mice showing gradual dissolution of Mg electrodes over two months post‐implantation (*n* = 4). Scale bars, 5 mm. Reproduced with permission [278]. Copyright 2023, AAAS. (d) Fully implantable, leadless, and battery‐free bioresorbable cardiac pacemaker. Left: schematic showing the device mounted on myocardial tissue. Center: structural layout of the system, including a W/Mg inductive coil and Si nanomembrane diode encapsulated in PLGA. Right: 3D CT images showing progressive bioresorption and complete disappearance of the pacemaker by day 46 (*n* = 3). Scale bars, 10 mm. Reproduced with permission [279]. Copyright 2021, Springer Nature.

Early demonstrations established that transient functionality can be achieved with biodegradable inorganic materials (Figure 10a) [258]. Silicon, silica, magnesium, and magnesium oxide undergo controlled hydrolysis in aqueous environments, gradually dissolving into biocompatible ions such as silicic acid or Mg^2^^+^ that are naturally processed by the body. Beyond these, other metals, including zinc, iron, molybdenum, and tungsten, have been explored as transient conductors and interconnects [257]. Their dissolution kinetics vary with local pH, temperature, and oxide formation, allowing the operational lifetime to be tuned from days to several months. These findings established a versatile materials palette for building resorbable electrodes, interconnects, and even transistors from fully degradable components.

Polymers represent another essential class of transient substrates and encapsulation materials (Figure 10b). Water‐soluble polymers such as poly(vinyl alcohol) (PVA) [259, 260] and poly(vinylpyrrolidone) (PVP) [261, 262] are widely employed as transient materials. These polymers dissolve or swell in aqueous environments, enabling physically triggered disintegration without generating acidic degradation products. Owing to their excellent film formability, biocompatibility, and optical transparency, PVA and PVP have been extensively utilized as substrates, encapsulants, and sacrificial layers in transient electronic systems. In addition, biodegradable polyesters such as poly(lactic acid) (PLA) [263, 264], poly(lactic‐co‐glycolic acid) (PLGA) [265, 266, 267], and poly(caprolactone) (PCL) [268, 269, 270, 271] offer tunable hydrolytic degradation profiles depending on composition, crystallinity, and processing. PLGA, in particular, has attracted extensive attention because its degradation rate can be precisely modulated by altering the lactide‐to‐glycolide ratio. Glycolide‐rich compositions promote rapid hydrolysis, whereas lactide‐rich systems degrade more slowly by limiting water uptake. More recently, biodegradable elastomers, including poly(glycerol sebacate) (PGS) [272, 273, 274], citrate‐based polymers [275, 276], and poly(l‐lactide‐co‐ε‐caprolactone) (PLCL) [277] have been reported. These combine softness, stretchability, and biodegradability, offering both mechanical compliance with tissues and controlled resorption. Such elastomers represent a critical step toward next‐generation transient platforms that can seamlessly interface with dynamic soft organs while disappearing after use.

Expanding upon these material innovations, a recent demonstration introduced a bioresorbable peripheral nerve stimulator capable of delivering transient electrical therapy for post‐operative pain management (Figure 10c) [278]. The system integrates biodegradable metals such as magnesium with PLGA encapsulation layers and soft elastomeric components, forming a fully implantable, wireless, and lead‐free architecture. Once implanted, the device provides localized electrical stimulation for several days, effectively blocking nociceptive signal transmission before safely dissolving into biocompatible byproducts. Wireless power transfer through an external control unit allows precise temporal modulation of stimulation without percutaneous connections or secondary surgeries. This approach exemplifies the translational potential of transient bioelectronics as short‐term therapeutic tools that bridge surgical recovery and natural healing, while minimizing infection risk and surgical burden.

Another promising direction for transient bioelectronics lies in fully implantable biodegradable systems designed for temporary organ support and physiological regulation. For instance, a transient cardiac pacemaker constructed entirely from biodegradable metals, polymers, and encapsulation layers demonstrated stable pacing performance for several days before dissolving harmlessly in vivo (Figure 10d) [279]. Beyond simple stimulation, recent advances have combined such transient implants with epidermal sensing modules to form closed‐loop therapeutic systems, in which the implanted device delivers stimulation while the external, skin‐mounted unit monitors physiological feedback in real time [280]. This distributed architecture enables autonomous and responsive treatment, paving the way for time‐limited therapeutic networks that operate seamlessly within the body and then disappear after fulfilling their function.

From a clinical perspective, transient electronics could address several unmet needs. A biodegradable pacemaker would be highly valuable for patients recovering from cardiac surgery, who may require pacing support only for a few weeks. Similarly, temporary neural stimulators could be used for post‐operative pain management, gradually disappearing once the acute phase has passed. Transient pressure or flow sensors could provide critical feedback following vascular grafting or gastrointestinal surgery, enabling early detection of complications without necessitating device removal. Collectively, these scenarios illustrate how transient electronics can reduce surgical burden and long‐term device–related risks by limiting hardware presence to the critical recovery window, while still providing essential functions such as pacing, neuromodulation, and physiological monitoring.​

Despite these advances, several critical challenges remain. Ensuring patient safety requires precise control over degradation rates, both material composition and device geometry. Strategies to trigger on‐demand dissolution through wireless, optical, or ultrasonic signals are being explored to improve reliability and control [281]. Equally important is the need to balance mechanical stability during operation with predictable degradation afterward, particularly for devices implanted in mechanically dynamic organs. Future efforts may also focus on integrating transient devices with drug delivery, tissue regeneration, or temporary modulation of neural and muscular activity.

In summary, transient electronics expand the design space of biointegrated systems by introducing the concept of time‐limited function followed by complete resorption. With advances in biodegradable metals, polymers, and elastomers, it is now possible to envision implantable devices that perform critical roles for days to months and then disappear without trace, eliminating the risks of surgical retrieval. As materials science, device engineering, and biomedical applications converge, transient electronics are poised to transform clinical practice by providing safer, less invasive, and more versatile therapeutic solutions.

## 8 Clinical Applications

The ultimate test of soft, stretchable, and tissue‐integrated bioelectronics lies in their translation to real clinical environments. In recent years, many of these technologies—initially demonstrated in research laboratories—have advanced to practical systems that directly benefit patients in intensive care, rehabilitation, and neuroengineering. Several representative examples illustrate how soft and wireless electronics are reshaping clinical practice.

**FIGURE 11:**
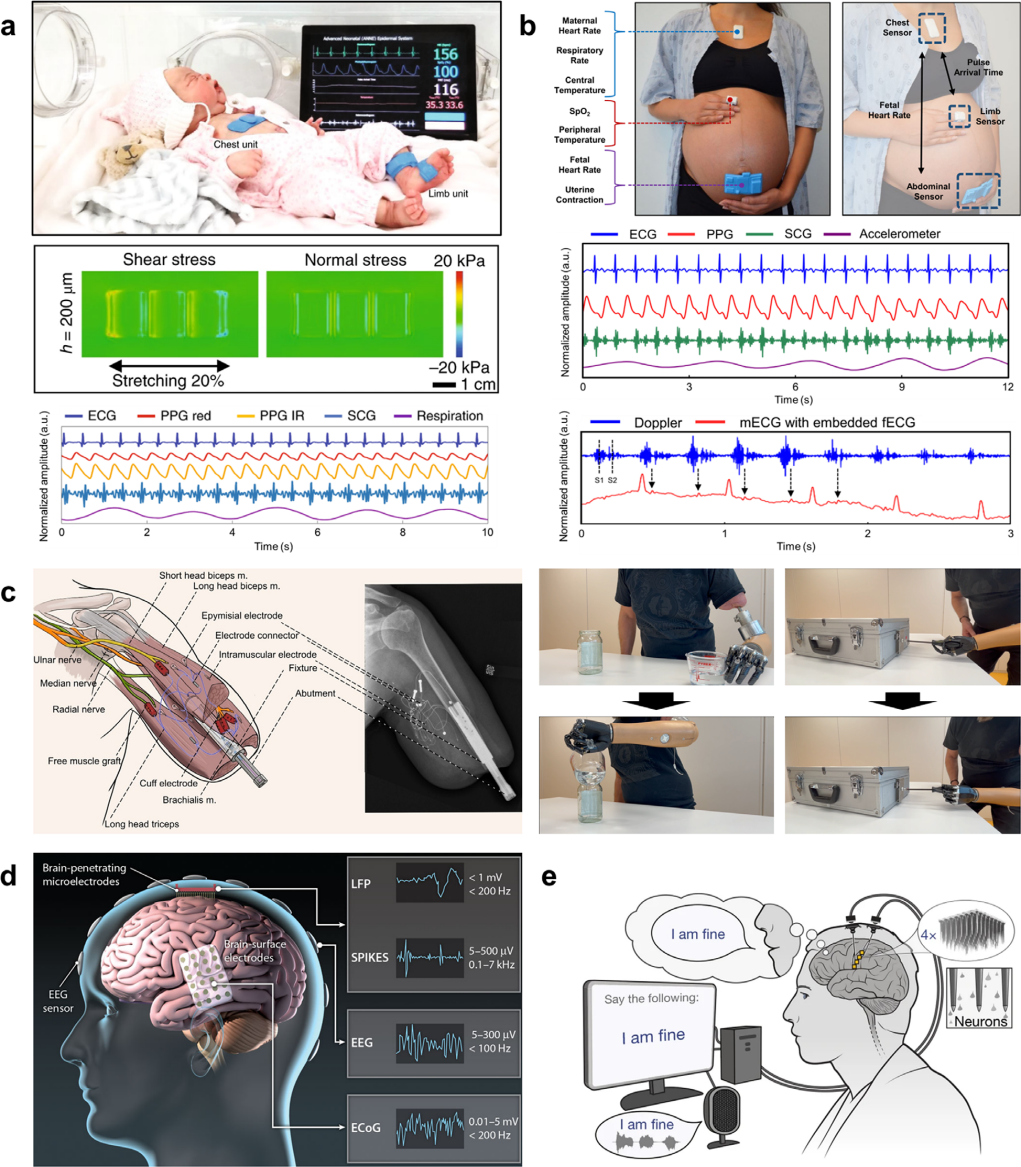
Clinical applications. (a) Damage‐free, skin‐interfaced biosensing for neonatal monitoring. Top: Wireless, skin‐conformal epidermal biosensors attached to a newborn infant for continuous vital‐sign monitoring. Middle: Finite‐element analysis shows low shear and normal stresses at the skin–device interface under 20% uniaxial stretching with a thin substrate, indicating mechanically benign contact. Bottom: Representative electrocardiography (ECG), photoplethysmography (PPG), seismocardiography (SCG), and respiration signals demonstrate stable, medical‐grade performance without skin damage. Reproduced with permission [283]. Copyright 2020, Springer Nature. (b) Comprehensive pregnancy monitoring with a network of soft, wireless sensors for high‐ and low‐resource settings. Top: The system integrates multiple sensors to measure fetal heart rate (FHR) and uterine contractions. Middle: Maternal vital signs obtained by the chest, limb, and abdominal sensors. Bottom Doppler‐derived FHR and EMG‐derived uterine contraction signals. Reproduced with permission [284]. Copyright 2021, NAS. (c) Prosthetic limb control via surgically created electro‐neuromuscular constructs with implanted electrodes. Left: schematic and X‐ray of the humanmachine interface. Right: patient using a neuromusculoskeletal arm prosthesis (4.5 DOF) performing daily tasks through neural‐networkbased proportional control. Reproduced with permission [285]. Copyright 2023, AAAS. (d) Brain–machine interfaces. Brain signal acquisition modalities; EEG, ECoG, local field potentials (LFPs), and spikes. Reproduced with permission [286]. Copyright 2013, AAAS. (e) Brain‐to‐voice neuroprosthesis enabling closed‐loop voice synthesis from intracortical neural activity in a participant with ALS. Reproduced with permission [290]. Copyright 2025, Springer Nature.

Soft epidermal biosensors have proven especially valuable in neonatal and maternal health monitoring, where conventional rigid electrodes and wired systems often cause discomfort or skin irritation [282]. As shown in Figure 11a, wireless and skin‐conformal devices enable continuous monitoring of vital signs such as heart rate, respiration, and temperature in newborn infants without damaging their delicate skin [283]. Unlike conventional systems that obstruct parental contact, these soft platforms preserve skin‐to‐skin bonding—an essential aspect of early development—while maintaining medical‐grade accuracy. A wireless monitoring platform powered through a bed‐integrated antenna was first demonstrated for neonatal intensive care [282], followed by rechargeable battery‐based devices that have now entered clinical use. Expanding on this concept, Figure 11b depicts a network of soft, flexible, and wireless sensors designed for comprehensive pregnancy monitoring [284]. These systems can simultaneously record maternal heart rate, uterine contraction, and fetal heartbeat, enabling noninvasive and continuous observation throughout pregnancy. Their successful deployment in both high‐ and low‐resource hospitals underscores the scalability and reliability of soft bioelectronics. Several of these systems have been commercialized through Sibel Health Inc., bridging academic research with real‐world healthcare.

Beyond physiological monitoring, soft biointerfaces have transformed the control of prosthetic limbs [152]. As shown in Figure 11c, surgically implanted electrodes that link peripheral nerves and muscles to robotic hands establish stable electro‐neuromuscular constructs, enabling amputees to perform complex, intuitive movements [285]. The combination of implanted neural interfaces and soft, skin‐mounted sensors provides a bidirectional communication pathway, transmitting both motor commands and sensory feedback. Such hybrid systems allow users to experience natural touch perception and proportional control, demonstrating the clinical maturity of bio‐integrated prosthetic technology.

A further frontier lies in Figure 11d, where brain–machine interfaces (BMIs) directly translate cortical activity into digital control of external systems [286]. Implantable ECoG and microelectrode arrays have enabled people with paralysis to manipulate robotic arms, computer cursors, or virtual keyboards purely through neural signals. A landmark clinical study demonstrated the first fully implanted BMI in a patient with late‐stage amyotrophic lateral sclerosis (ALS), achieving autonomous home communication via subdural electrodes and a wireless chest transmitter for more than six months [287]. The successful long‐term operation of this system highlights the transition of BMIs from controlled laboratory environments to daily home use. While such translation introduces new ethical and safety considerations, it underscores the transformative potential of neural interfaces—not only as assistive communication tools but also as emerging therapeutic systems for motor and cognitive rehabilitation in neurological disorders such as Parkinson's [177, 178] and Alzheimer's disease [180].

Collectively, these clinical examples demonstrate that soft and tissue‐integrated electronics are beginning to redefine the interface between humans and machines. Their shared foundations—mechanical softness, conformability, wireless functionality, and biocompatibility—address long‐standing barriers in conventional medical instrumentation. Remaining challenges include ensuring operational stability under physiological conditions, establishing safety standards, and developing regulatory frameworks for long‐term use. Nevertheless, the fusion of skin‐mounted sensors, implantable stimulators, and intelligent analytics promises a new generation of closed‐loop healthcare systems that operate harmoniously with the body. Through these advances, soft bioelectronics are poised to transform clinical medicine by enabling safer, less invasive, and more human‐centered therapeutic solutions.

## 9 Conclusions and Outlook

The era when progress in bioelectronics could be defined merely by making devices thinner, softer, or more stretchable has come to an end. The next stage demands a deeper convergence of materials science, mechanical design, and physiology—not just to make electronics mechanically compatible with the body, but to ensure they functionally harmonize with living processes. True bio‐integration means developing systems that can communicate bidirectionally with biological signals, adapt dynamically to physiological changes, and even support healing and regeneration. Achieving this paradigm shift requires an integrated understanding of how electronic and biological systems can coexist and coevolve within the same physical and functional domain.

Realizing such systems will require close collaboration not only between engineers, materials scientists, and clinicians but also with industry partners. Engineers must go beyond device physics to understand the biological and clinical realities of medical applications. Practical devices must operate safely and stably over long periods, deliver clinically reliable data, offer intuitive usability for patients and healthcare professionals, and meet cost and scalability requirements for industrial production.

At the same time, the very softness and tissue affinity of these systems open completely new frontiers—enabling the detection and treatment of physiological phenomena that rigid devices could never access. Skin‐ and organ‐conformal sensors, stimulators, and feedback interfaces will illuminate previously invisible biological processes and offer unprecedented therapeutic options. Even if clinical implementation remains a long‐term goal, such research challenges must be actively pursued by engineers. The mission of engineering has evolved beyond building better devices—it now involves redefining the relationship between humans and technology.

As bio‐integrated electronics advance, the management and interpretation of the vast biological data they generate will become increasingly critical. Artificial intelligence (AI)‐based analytics provide the most realistic and powerful approach for extracting meaningful insights from continuous, multimodal physiological data streams. However, this development introduces new responsibilities: ensuring data privacy, security, and ethical use will be as important as the accuracy of sensing itself. The integration of AI with bioelectronics must therefore proceed with careful attention to transparent data governance, cybersecurity, and patient consent frameworks, ensuring that the benefits of these technologies are realized safely and equitably.

Ultimately, the future of bio‐integrated electronics lies not in merely mimicking the structure or mechanics of living systems, but in understanding and complementing their functions. As materials science, device engineering, and biological research continue to merge, electronic systems will evolve from external tools into symbiotic partners that support health, healing, and the human experience itself.

## Funding

JSPS KAKENHI Grant Number JP25K21548 and JP22K21343.

## Conflicts of Interest

The authors declare no conflicts of interest.

## Data Availability

The data that support the findings of this study are available from the corresponding author upon request.
